# Syncopation, Body-Movement and Pleasure in Groove Music

**DOI:** 10.1371/journal.pone.0094446

**Published:** 2014-04-16

**Authors:** Maria A. G. Witek, Eric F. Clarke, Mikkel Wallentin, Morten L. Kringelbach, Peter Vuust

**Affiliations:** 1 Faculty of Music, University of Oxford, Oxford, United Kingdom; 2 Department of Psychiatry, Warneford Hospital, University of Oxford, Oxford, United Kingdom; 3 Center of Functionally Integrative Neuroscience, Aarhus University Hospital, Aarhus, Denmark; 4 Center for Semiotics, University of Aarhus, Aarhus, Denmark; 5 The Royal Academy of Music, Aarhus/Aalborg, Denmark; VU University Amsterdam, Netherlands

## Abstract

Moving to music is an essential human pleasure particularly related to musical groove. Structurally, music associated with groove is often characterised by rhythmic complexity in the form of syncopation, frequently observed in musical styles such as funk, hip-hop and electronic dance music. Structural complexity has been related to positive affect in music more broadly, but the function of syncopation in eliciting pleasure and body-movement in groove is unknown. Here we report results from a web-based survey which investigated the relationship between syncopation and ratings of wanting to move and experienced pleasure. Participants heard funk drum-breaks with varying degrees of syncopation and audio entropy, and rated the extent to which the drum-breaks made them want to move and how much pleasure they experienced. While entropy was found to be a poor predictor of wanting to move and pleasure, the results showed that medium degrees of syncopation elicited the most desire to move and the most pleasure, particularly for participants who enjoy dancing to music. Hence, there is an inverted U-shaped relationship between syncopation, body-movement and pleasure, and syncopation seems to be an important structural factor in embodied and affective responses to groove.

## Introduction

What is it about certain kinds of music that makes us want to move, and why does it feel good? Few contexts make the pleasurable effects of music more obvious than the dance club. The ways in which bodies synchronise to regular yet rhythmically complex beats are perhaps the most overt expressions of music-induced pleasure. While, more broadly, the link between body-movement and affect has received significant empirical support [1]–[4], in accordance with embodied theories of cognition [5], [6], we know little about how music induces a desire for bodily movement. Behaviourally, groove has been described as a musical quality associated with body-movement and dance [7]–[10], often occurring in response to musical genres such as funk, soul, hip-hop and electronic dance music. Structurally, this music is often characterised by syncopation [11]–[14]. However, the role of syncopation in promoting pleasurable sensorimotor synchronisation remains unclear. In this study, we investigated the relationship between syncopation in groove rhythms and feelings of wanting to move and pleasure by asking participants to rate their groove-related experiences via a web-based survey.

Pleasure and emotional responses to music have been linked to expectation and anticipation [15]–[19]. For example, music's ability to send shivers down the spine is suggested to result from the violation of structural expectations [20]–[25]. Such musically induced ‘chills’ have also been shown to correlate with activity in the reward network of the brain [26], [27]. Despite both pleasure [16], [26], [27] and sensorimotor synchronisation [28], [29] being proposed as factors in music's evolutionary origin, few have studied the pleasure of sensorimotor synchronisation. It has been shown that the more people experience a desire to move to music, the more they enjoy it [10]. Furthermore, babies exhibit positive affect when being bounced to rhythmically regular music [30]. Rhythmic entrainment, i.e. the process by which attention becomes coupled with another rhythmic stimulus [31]–[35], often overtly expressed through sensorimotor synchronisation [36]–[39], has been suggested to tap into affective mechanisms [30], [40], [41]. For example, it is thought that entrainment and sensorimotor synchronisation evoke positively valenced experiences through the mechanism of emotional contagion [40]–[42]. When overtly (or covertly) synchronising to music in a social context, the emotional states of one person may be transferred to another, via shared attention to time and dynamics. However, what it is about music that offers a pleasurable desire to move is unclear. Most researchers studying musical affect have largely focused on melodic and harmonic structures, instead of rhythm [20], [43]. Recently, Keller and Schubert [44] showed that melodies which violate rhythmic expectations were rated as more enjoyable and ‘happier’ than rhythmically predictable melodies, suggesting that rhythmic complexity is an important factor in understanding why people enjoy listening to music [45]–[47].

In a classic study, Berlyne [48] proposed that an inverted U-shaped curve (also called the Wundt curve [49]) reflects a general relationship between aesthetic appreciation and structural complexity in art. According to this relationship, increasing complexity correlates positively with liking, arousal and pleasure up to an optimal point, after which a further increase in complexity reverses the effect. The theory was first empirically demonstrated for music by Heyduk [50] and was subsequently appropriated for ratings of subjective complexity in popular music [45]–[47]. However, what constitutes the optimal level of complexity depends on musical context [46] personality [51], genre and listening preferences [52]. It is likely that culture also governs affective responses to complexity in music, since the music of different cultures can vary in levels and expressions of complexity [53], [54].

The relationship between musical complexity and affect may also depend on the type of response associated with a genre. In groove, responses are largely rooted in sensorimotor synchronisation and dance [7]–[10]. Wanting to move is reported as the most consistently and robustly defined subjective experience in response to groove [7], [9], [10]. Although there are stylistic differences in genres associated with groove, most groove-induced dance is rhythmically periodic and synchronised to the metre. Using motion-capture, Toiviainen et al. [36] showed that although both higher and lower metric levels were expressed in different body-parts during spontaneous dancing to instrumental blues, the quarter-note (main pulse) and half-note were the most salient. Janata et al. [10] related movement-induction more closely to positive affect by showing that the extent to which participants enjoyed the music and felt ‘in the groove’ also defined their experiences of groove-related desire for body-movement. However, it is still unclear how structural components of music associated with groove elicit pleasure.

In a study that investigated the relationship between ratings of wanting to move and structural and acoustic properties of music associated with groove, Madison et al. [9] found that beat salience and event density (sub-beat variability) correlated positively with ratings. They did not find an effect of microtiming, which has been the focus of many groove studies [55]–[57]. In fact, a later study showed that microtiming decreased liking and the desire to move [58]. Microtiming is often referred to as deviations from rhythmic isochrony on a millisecond level, often expressed in performance [59]–[62], but also purposefully composed by some contemporary producers [63], [64]. Compared to microtiming, syncopation is a more large-scale, composed form of rhythmic complexity, broadly thought of as a shift of rhythmic emphasis from metrically strong to metrically weak beats [65], [66]. Syncopation characterises many genres associated with groove, e.g. funk [11], electronic dance music [14], jazz [13] and hiphop [12]. Another important structural feature of these genres is repetition [11], [14], [56], [67], [68]. Because of repetition, any microtiming or syncopation is experienced cyclically [11]. It is likely that this repetitiveness contributes to the strong propensity towards sensorimotor synchronisation associated with groove, since continuous synchronisation requires predictability [9], [10], [68]. However, it is unclear to what extent syncopation within the repeated patterns influences the experience of groove.

Relating directly to the link between rhythm and body-movement in groove, a growing body of research shows that rhythm perception is associated with activity in areas of the brain known to be involved in motor perception and action, such as premotor cortex, supplementary motor area, cerebellum and the basal ganglia, and that activity in these regions is modulated by rhythmic complexity [69]–[75]. Specifically for groove, Stupacher et al. [8] found that movement induction in response to music associated with groove was mediated by motor systems in ways that were modulated by musical training [10]. Furthermore, a study using electroencephalography has shown that the firing patterns of neurons in the brain entrain to the metric periodicities in auditory rhythm, even when some of the acoustic information about the periodicities is missing [76].

Humans' ability to perceive regularity in rhythm, even when the rhythm itself is not uniformly regular, relies on the mechanism of metre perception. Involving the perception of regularly alternating strong and weak accents, metre in music forms nested levels of isochronous pulses that can be hierarchically differentiated based on their accentual salience [35], [77]. More often than as a source of affect in music, rhythmic complexity has been used in empirical research to reveal the mechanisms underpinning metre perception [78]–[82]. While some have systematically varied the degree of rhythmic complexity as a factor in musical pattern recognition [83], others have been interested in how well rhythmic properties can model human judgements of complexity [81].

Syncopation is one of the most studied forms of rhythmic complexity in music [65], [79], [84]–[88]. It can be defined as a rhythmic event that violates listeners' metric expectations [65], [79], [86], [88]. Longuet-Higgins and Lee [65] proposed a computational index for calculating the strength of a syncopation, using a hierarchical model of metric salience. They define syncopation as a note on a metrically weak accent preceding a rest on a metrically strong accent, and their model computes the degree of syncopation based on the difference in metric weights between the note and the rest that constitute the syncopation. A number of researchers have used syncopation in modelling rhythm and metre perception. Some have investigated the extent to which syncopation affects metre perception and the ability to entrain [85], [89]–[92]. Fitch and Rosenfeld [85] showed that high degrees of syncopation prevented the perception of metre and reduced the ability to synchronise finger-tapping. Others have used expectancy violation in syncopation as a tool for perceptually validating metric models [79], [86], [93], [94]. In a study comparing 32 different computational measures of rhythmic complexity, Thul and Toussaint [84] found that measures of syncopation outperformed other measures in explaining the behavioural data from four separate studies. The data comprised of judgements regarding perceptual, metric and performance complexity of rhythmic patterns. It was found that models of syncopation better explained the variability in these judgements, compared to for example standard deviation and entropy (i.e. the degree of uncertainty in a random sample, from an information theory perspective [84], [95], [96]). Syncopation therefore appears to be a more appropriate predictor of perceived rhythmic complexity.

Despite the ubiquity of syncopation in music associated with groove, its effects on affective and sensorimotor responses have remained largely unexplored. Since: a) structural complexity is related to positive affect [45], [46], [48], b) syncopation is a common form of structural complexity in music associated with groove [11]–[14], and c) groove elicits a pleasurable drive towards body-movement [9], [10], we investigated the extent to which syncopation can explain the desire to move and feelings of pleasure in groove. That is, if structural complexity is related to positive affect, then it is possible that the positive affect associated with groove is related to its structural complexity. And since syncopation is a common form of complexity in music associated with groove and positive affect in groove is related to a desire for body-movement, syncopation is a likely candidate for explaining the link between pleasure, desire for movement, and groove. Specifically, we hypothesised that there would be an inverted U-shaped relationship between degree of syncopation in groove rhythms and ratings of wanting to move and experience of pleasure, in accordance with Berlyne's theory [48]. Since the body-movements associated with groove-based music are primarily entrained to the metre [36], it is likely that the desire to move is maximised by syncopated rhythms that optimise such sensorimotor synchronisation. Hence, rhythms that are so syncopated that they disrupt the metre should be less likely to elicit the desire to move or feelings of pleasure. Conversely, rhythms with little or no syncopation should be unlikely to induce body-movement or pleasure since they lack the structural complexity that is both related to pleasure in music more generally and that characterises the rhythmic structure of music associated with groove specifically. Rather, rhythms with medium degrees of syncopation should be most likely to elicit body-movement and pleasure, since such rhythms include enough rhythmic complexity to stimulate responses, but not so much as to prevent entrainment.

In order to test our hypothesis, participants were invited to complete a web-based survey which involved listening to a series of synthesised drum-breaks which varied in their degree of syncopation, and to rate how much these made them want to move and how much pleasure they experienced. We also investigated whether the musical background of listeners [8], [52], [93] affected the desire for body-movement and feelings of pleasure, based on participants' self-reported levels of musical training, familiarity with groove-based genres, and frequency and enjoyment of dancing.

## Methods

### Ethics Statement

This study investigates subjective experiences of music via a web-based survey. The ethical committee to which the majority of the authors of the present paper report is the Central Denmark Region Committees on Health Research Ethics. According to their Act on Research Ethics Review of Health Research Projects (Act 593 of 14 July 2011, section 14.1), only health research studies shall be notified to the Committees. Our study is not considered a health research study (section 14.2) and therefore did not require ethical approval nor written/verbal consent, regardless of participants' age. When recruited, participants were informed that their responses would be used for research purposes. Participants were anonymised and no IP addresses were collected or stored. They were free to exit the survey at any time, and provided with an email address at the end of the survey to which they could address any questions or concerns.

### Participants

Sixty-six participants aged between 17 and 63 (Mean = 30.14, SD = 10.79), from countries in Europe, Oceania, Africa, America and Asia, were recruited to complete the survey on a voluntary basis, through opportunity sampling. The questionnaire was in English only, and although there might have been language issues for those whose first language was not English, we assume that these influences were minor. Furthermore, given that we primarily investigated within participant differences, any false positive effect of language would be likely be cancelled out.

A questionnaire recorded details on further demographics. Participants were defined according to musical training (musicians >8 years of training, non-musicians <4 years of training), groove familiarity and dance experience (according to Likert scales). Table 1 reports group sizes. Nine participants were excluded from analyses involving musical background, since they could neither be categorised as musicians, nor non-musicians. See Text S1 for more details on musical background categorisation.

**Table 1:** Musical background group size.

Musical Training		Groove Familiarity		Dance Experience	
Musician	Non-Musician	Groove-Enjoyer	Non-Groove-Enjoyer	Dancer	Non-Dancer
22	35	39	18	37	20

Notes: N for each category of musical background (total N = 57). See Text S1 for categorisation and inclusion criteria. Sex was only recorded for 42 participants (20 females, 22 males).

Participants were also asked to confirm whether they used good quality headphones or sound system for the experiment. We did not ask whether they used headphones or sound system. 54 participants reported being able to use good quality headphones or sound system, while only 12 reported not being able to do so. A 2×3×2 ANOVA, with rating question (movement and pleasure), syncopation degree (Low, Medium and High, see later analyses for description of categorisation) and audio quality (‘good’ or ‘not good’) as independent variables showed that there was no significant effect of audio quality (F(1, 64)  = .01, *p* = .961), nor any interactions with rating question (F(1, 64)  = .31, *p* = .581) or syncopation degree (F(1, 64) = .77, *p* = .465).

### Stimuli

The stimuli consisted of 50 drum-breaks programmed using a synthesised drum-kit (bass-drum, snare-drum and hihat) in GarageBand 5.1 (Apple, Inc.). Each break consisted of a two-bar phrase looped four times in 4/4 time at 120 bpm, each break lasting 16 seconds. Syncopations occurred in a number of configurations within the bass- and snare-drum parts, while the hihat maintained a constant quaver pulse (see Figures S1–S4 for transcriptions of all 50 drum-breaks).

The degree of syncopation was calculated using an index of syncopation broadly modelled on that of Longuet-Higgins and Lee [65], but using a less hierarchical model of metre and additional instrumental weights to take account of the drum-breaks' polyphonic character (see Text S2 and Figures S5–S7 for detailed description of the index). Thus, our definition of syncopation depended not only on differences in metric weights between rests and notes, but also between notes played on different instruments of the drum-kit. For example, a snare-drum on a metrically weak accent followed by a bass-drum on a metrically strong accent would constitute a syncopation, and the degree of syncopation would depend on the difference between weight of the notes played by the two drum instruments.

In addition, a measure here called the ‘joint audio entropy’ of the drum-breaks was computed, in order to compare the performance of the syncopation index with other models of complexity. Joint entropy is a measure of the uncertainty in two or more discrete variables [84], [95]. Here, we computed the joint entropy of the audio wave data, thus measuring the probability of each wave sample occurring on the basis of the distribution of the wave data as a whole (see Text S3 for a detailed description of the measure). Entropy acted as a purely acoustic measure of complexity, to be compared with the more behaviourally defined measure of syncopation.

Out of the 50 drum-breaks, 34 were transcribed from real funk tracks. Two drum-breaks were transcribed from drum-kit groove templates from Garageband 5.1 (Apple, Inc.). The remaining 14 drum-breaks were specifically constructed for the experiment in order to increase the spread of syncopation at both ends of the spectrum (i.e. weakly syncopated, and very syncopated) and to control for the number of onsets, since event density has been shown to affect groove responses [9]. None of the drum-breaks included any microtiming. Pearson's correlations showed that syncopation did not correlate significantly with total number of onsets (*r* = .092, p = .526). There was a close-to-significant small correlation between syncopation and joint audio entropy (*r* = .259, p = .067), which may have been caused by both measures representing complexity, albeit based on different methods of computation: A syncopated pattern might be described in terms of uncertainty (unexpected note onsets), but uncertainty can be expressed in other ways than syncopation (e.g. microtiming). Nonetheless, in the context of this study, syncopation was treated as statistically independent from both total number of onsets and entropy.

### Procedure

Participants were invited to visit a webpage to take part in the survey. After completing the demographics questionnaire (Figure S8), they heard two drum-breaks, which were not part of the experiment, during which they were asked to adjust the volume on their computers to an enjoyable but comfortable level. Then each experimental drum-break was presented individually, in a fully randomised order. During each drum-break, participants were asked to rate:

- To what extent does this rhythm make you want to move?
- How much pleasure do you experience listening to this rhythm?

See Figure S9 for an image of the survey. Ratings were recorded on 5-point Likert scales (from 1 =  not at all/none, to 5 =  very much/a lot). Participants were able to proceed to the next drum-break only after they had heard the whole of the previous drum-break. The whole experiment lasted 15-20 minutes.

## Analysis

Although wanting to move and pleasure are strongly connected in groove [10], it was decided to treat these measures separately in order to test the extent to which they are linked and how they interact with other variables, such as musical background. In the analyses where we were interested in such effects (i.e. *Model Comparisons* and *Musical Background and Interactions*), pleasure and movement-desire were treated as two separate levels of an independent variable, ‘rating question’. The close relationship between pleasure and movement in groove did not cause co-linearity/orthogonality problems in these analyses, since the variable ‘rating question’ represented two categories as opposed to covariates (i.e. the actual data points were not entered into the statistical model). In all other analyses (*i.e. Individual Regressions* and *Predictor Contributions*), statistical tests were conducted in parallel, separately for pleasure and wanting to move. In other words, here the two rating questions operated as separate dependent variables.

### Individual Regressions

As a first indication of the relationship between movement- and pleasure-ratings and syncopation and joint audio entropy, each participant's ratings were first regressed against the drum-breaks with the two complexity measures as predictors. Of primary interest was whether the putative relationships were linear or quadratic. Thus, both a straight line and a parabola were fitted to each participant's ratings as indexed by the descriptors.

### Model Comparisons

In order to test whether these observations were statistically significant, a three-way within-subjects ANOVA was performed on the adjusted R^2^ value for each subject's regressions of ratings as the dependent variable; and predictor (syncopation vs. entropy), rating question (pleasure vs. wanting to move), and model (linear vs. quadratic) as independent variables. Due to the already high number of variables in this ANOVA, we decided not to increase its complexity even further by adding between-subject variables as well. See *Musical Background and Interactions* for analysis of between-subjects effects.

It is important to use the adjusted R^2^ when comparing models with different numbers of terms, such as when comparing linear (one-term) with quadratic (two-term) models. Compared to the normal R^2^, which represents the amount of variance in the sample that can be accounted for by the model, the adjusted R^2^ represents the amount of variance had the model been derived from the wider population from which the sample is taken. Importantly, it also includes a penalty for models with higher polynomials: adding terms to a regression increases the R^2^, but at the expense of a more complex model. The outcome of the ANOVA indicates which model fitted ratings best across participants, depending on whether the drum-break was considered in terms of syncopation or entropy, and with regard to the desire to move or feelings of pleasure. However, it does not indicate whether the best-fitting models are negative or positive.

### Predictor Contributions

In order to test the relative contribution of the two predictors and statistically determine whether the model had a negative or positive fit, a multiple regression analysis was performed on mean ratings for each drum-break, using only quadratic models. The analyses were performed separately for each rating question, using multiple regression with the forward stepwise method. The predictors were transformed into linear representations of quadratic models, by centring (subtracting the mean) and squaring.

### Musical Background and Interactions

Although the regression analysis differentiates between the fit of quadratic and linear models to the relationships between predictors and ratings for each drum-break, it ignores the effects of participants' musical backgrounds. Furthermore, any interactions with musical background and rating question are not addressed (e.g. whether participants rated wanting to move and feelings of pleasure differently depending on the level of syncopation, joint audio entropy and/or musical background). To investigate the effects of musical background and possible interactions, the syncopation and entropy predictors were transformed from continuous variables to three-level factors of Low, Medium and High, with almost equal numbers of drum-breaks' ratings in each category (see Table S1 for indexing of predictor values according to categories), and analysis performed in two separate 2×3×2×2×2 ANOVAs.

## Results

Overall, the results of our study support the hypothesis that there is an inverted U-shaped relationship between degree of syncopation and ratings of wanting to move and feelings of pleasure. The measure of syncopation (S) was a significant predictor of participants' ratings, while the joint audio entropy measure (JAE) was found to be a poor predictor, and ratings were particularly affected by participants' frequency and enjoyment of dancing. In what follows, these main results are described in detail, and a number of other results reported. For an empirical validation of the index used, see Text S4. Additional statistical tests were performed for ratings using only ‘real’ drum-breaks, excluding experimenter-composed stimuli, in order to control for the potential ‘unusualness’ of the experimenter-composed drum-breaks. Analysis and results are reported in Text S5. Another analysis, which can be found in Text S6, reports the effects of musical background on the U-shape vertex.

### Individual Regressions

**Figure 1:**
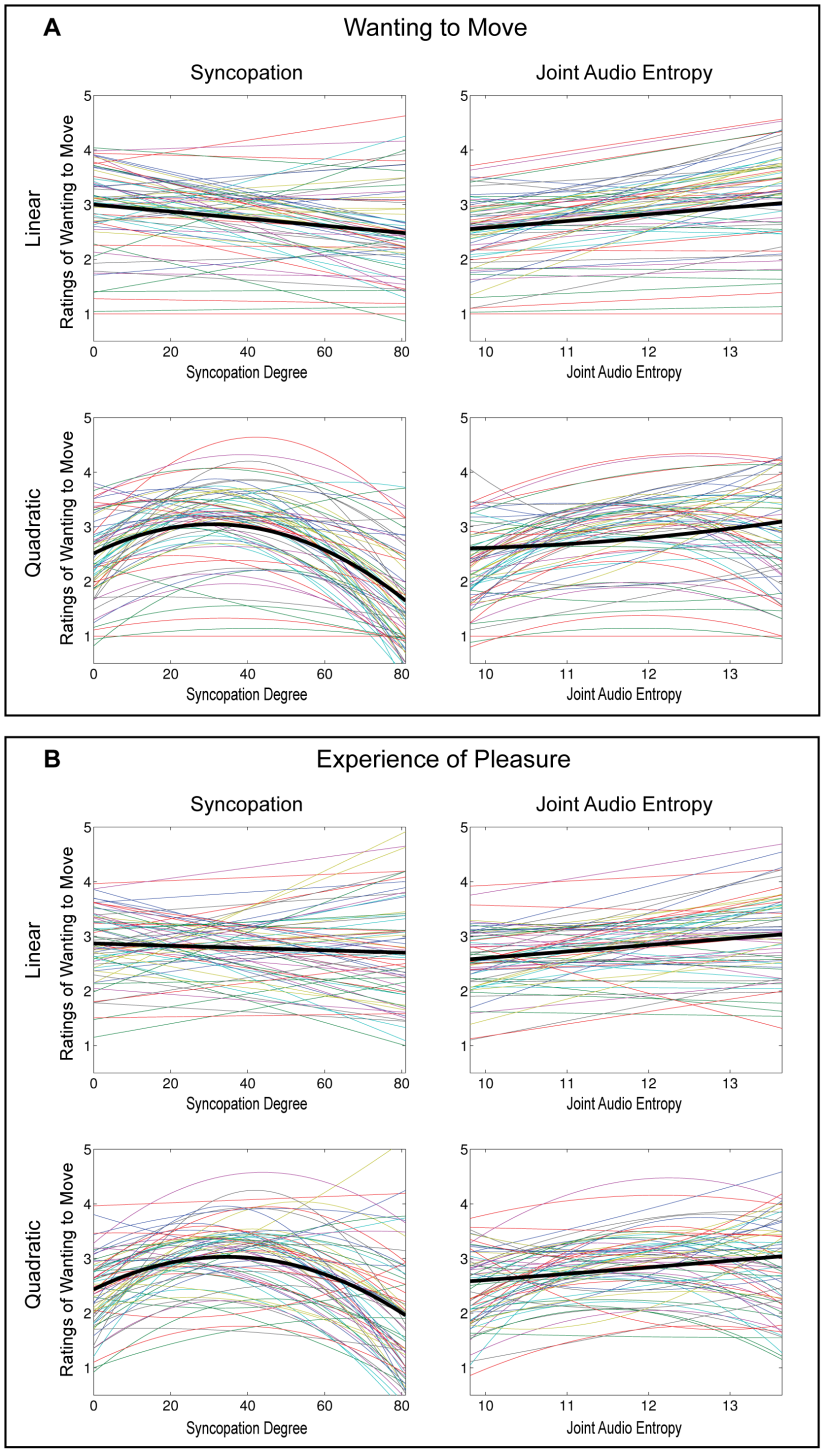
Individual regressions. Linear and quadratic regressions of stimuli predictors – syncopation and joint audio entropy – for ratings of A: wanting to move. B: experience of pleasure. Coloured lines represent individual subjects' regression fit with ratings; thick black line represents mean regression fit across subjects. Syncopation X axes  =  stimuli's syncopation degree, min 0 – max 81, calculated according to index of syncopation described in Text S2. Joint Audio Entropy X axes  =  stimuli's joint audio entropy, min 9.81 – max 13.65, calculated according to function described in Text S3. Y axes  =  Likert scale ratings, min 1 (not at all/none) – max 5 (very much/a lot).

Figure 1 shows each participant's ratings fitted with linear and quadratic regressors, for predictors and rating questions separately. The black line represents the fit to mean ratings. The figure shows that the quadratic models are more convergent across participants for S, and that the difference between linear and quadratic fit is less pronounced for JAE.

### Model Comparisons

**Figure 2:**
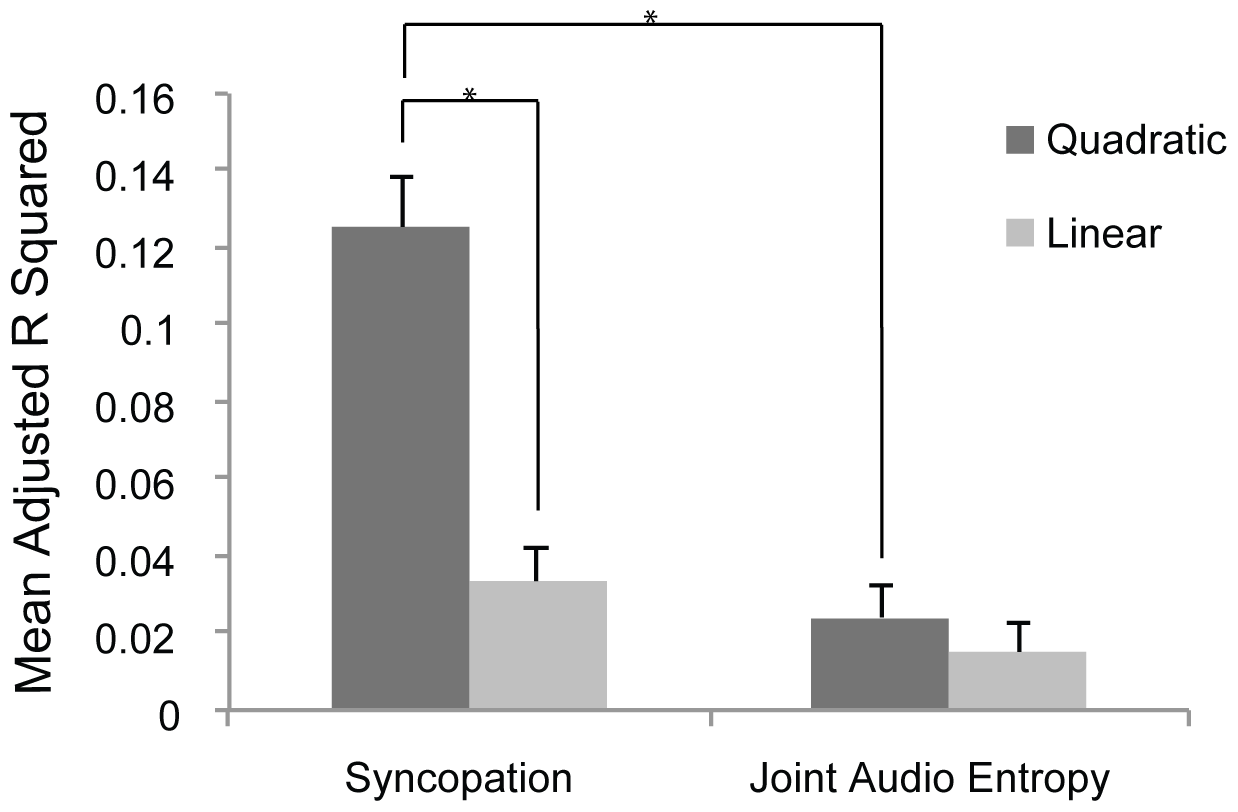
Model and predictor interaction. Interaction between models (quadratic and linear) and predictors (syncopation and joint audio entropy) on individual subjects' adjusted R^2^. Error bars  =  standard error. *Alpha adjusted *p*<.01.

The within-subjects ANOVA performed on adjusted R^2^ values for each subject's linear and quadratic regression confirmed these observations, showing a significant main effect of predictor and model, but not of rating question (Table 2). There was also a significant interaction between predictor and model. This interaction was followed up with paired t-tests, corrected for multiple comparisons (Table 3, Figure 2), which showed that quadratic models fitted the data better than linear models for S but not for JAE. Furthermore, the quadratic model showed a better fit with S than JAE. Thus, wanting to move and pleasure is related to degree of rhythmic complexity in groove in a U-shaped way and is better described by syncopation than by joint audio entropy.

**Table 2:** Main effects and interactions of predictor, model and rating question on adjusted R^2^.

Main Effect/Interactions	F	p
Predictor	18.56	<.001
Model	64.91	<.001
Rating Questions	2.00	.162
Predictor x Model	42.04	<.001
Rating Question x Model	2.26	.138
Predictor x Rating Question	2.07	.155

Notes: Degrees of freedom(error)  = 1(65).

**Table 3:** Paired contrasts for predictor and model on adjusted R^2^.

Contrasts		Mean	SE	t	p
Quadratic vs. Linear	Syncopation	0.09	0.01	7.65	<.001*
	Joint Audio Entropy	0.01	0.004	2.38	.020
Syncopation vs. Joint Audio Entropy	Quadratic	0.10	0.02	6.08	<.001*
	Linear	0.02	0.01	1.35	.183

Notes: Effect of models (quadratic and linear) and predictors (syncopation and joint audio entropy) on adjusted R^2^. Degrees of freedom  = 65. *Alpha adjusted *p*<.01.

### Predictor Contributions

The multiple regression on average ratings showed that, for both wanting to move and experience of pleasure, only the syncopation predictor contributed significantly to the U-shaped model. R^2^ = .3474 for wanting to move, and .4267 for pleasure, which were both significant (*F*(1,48)  = 25.56, *p*<.001, and *F*(1,48) = 35.73, *p*<.001, respectively). Table 4 reports the coefficients, which were all negative. Thus, the U-shaped model was confirmed to be inverted, and the rating variance explained by joint audio entropy did not significantly add to the variance already explained by syncopation.

**Table 4:** Regression coefficients of syncopation for ratings.

	Wanting to Move			Experience of Pleasure		
	*B*	*SE B*	*β*	*B*	*SE B*	*β*
Constant	3.076	0.083		3.047	0.059	
Syncopation	−.001	<.001	−.589*	−.001	<.001	−.653*

Notes: Wanting to move *r* = .5894, R^2^ = .3474; experience of pleasure *r* = .6532, R^2^ = 4267. * *p*<.001.

### Musical Background and Interactions

**Figure 3:**
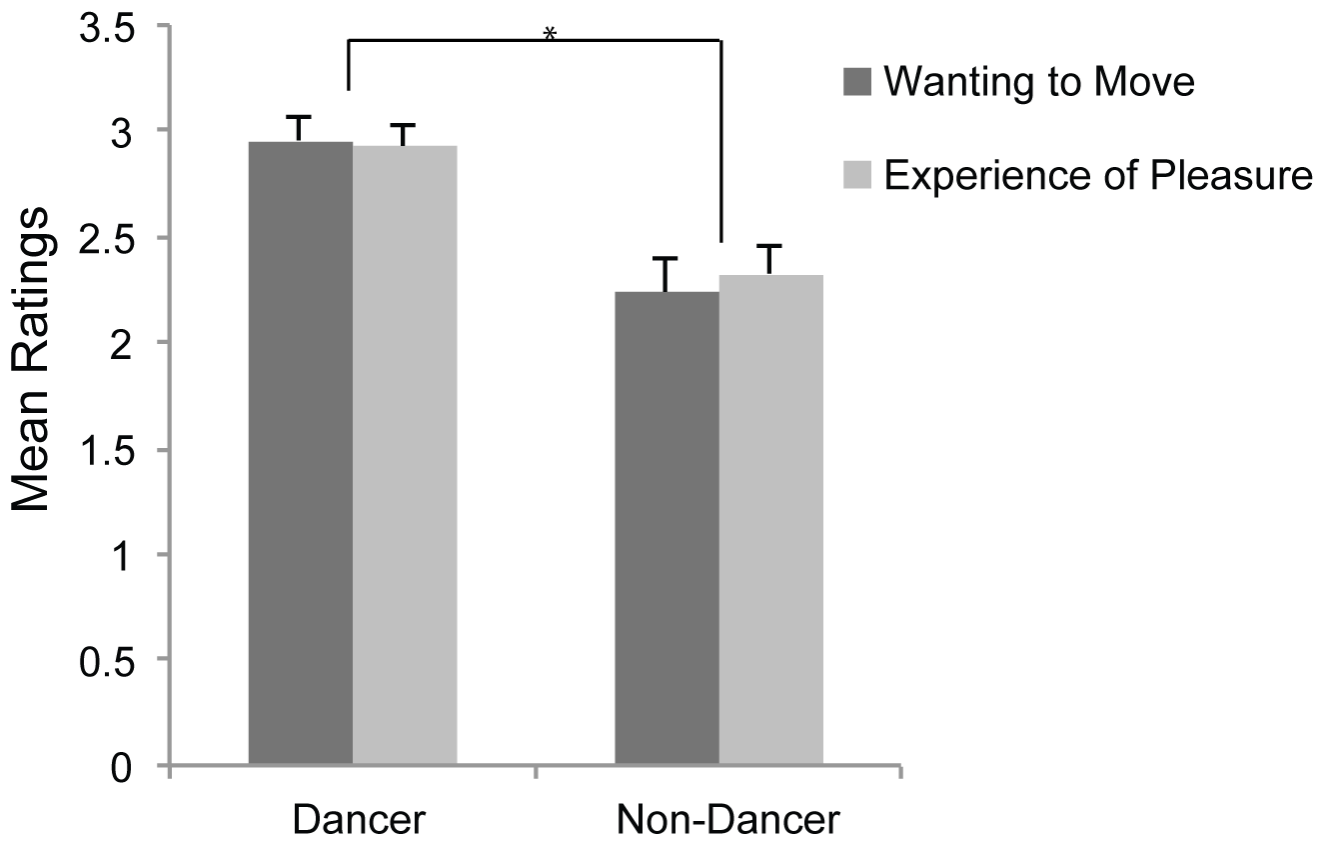
Effect of dancing experience. Effect of dancing experience on ratings of wanting to move and experience of pleasure. Error bars  =  standard error. *p<.01.

The ANOVA using Low, Medium and High levels of syncopation showed a significant between-subjects effect of dancing experience (*F*(1, 49)  = 13.53, *p* = .001), specifically that dancers rated drum-breaks as more movement- and pleasure-inducing than non-dancers (Figure 3). The effect of musical training approached significance (*F*(1, 49)  = 3.64, *p* = .062, musicians Mean  = 2.43, S.E = .14, non-musicians Mean  = 2.77, SE  = .11), but there was no main effect of groove familiarity (*F*(1, 49)  = .70, *p* = .439).

**Figure 4:**
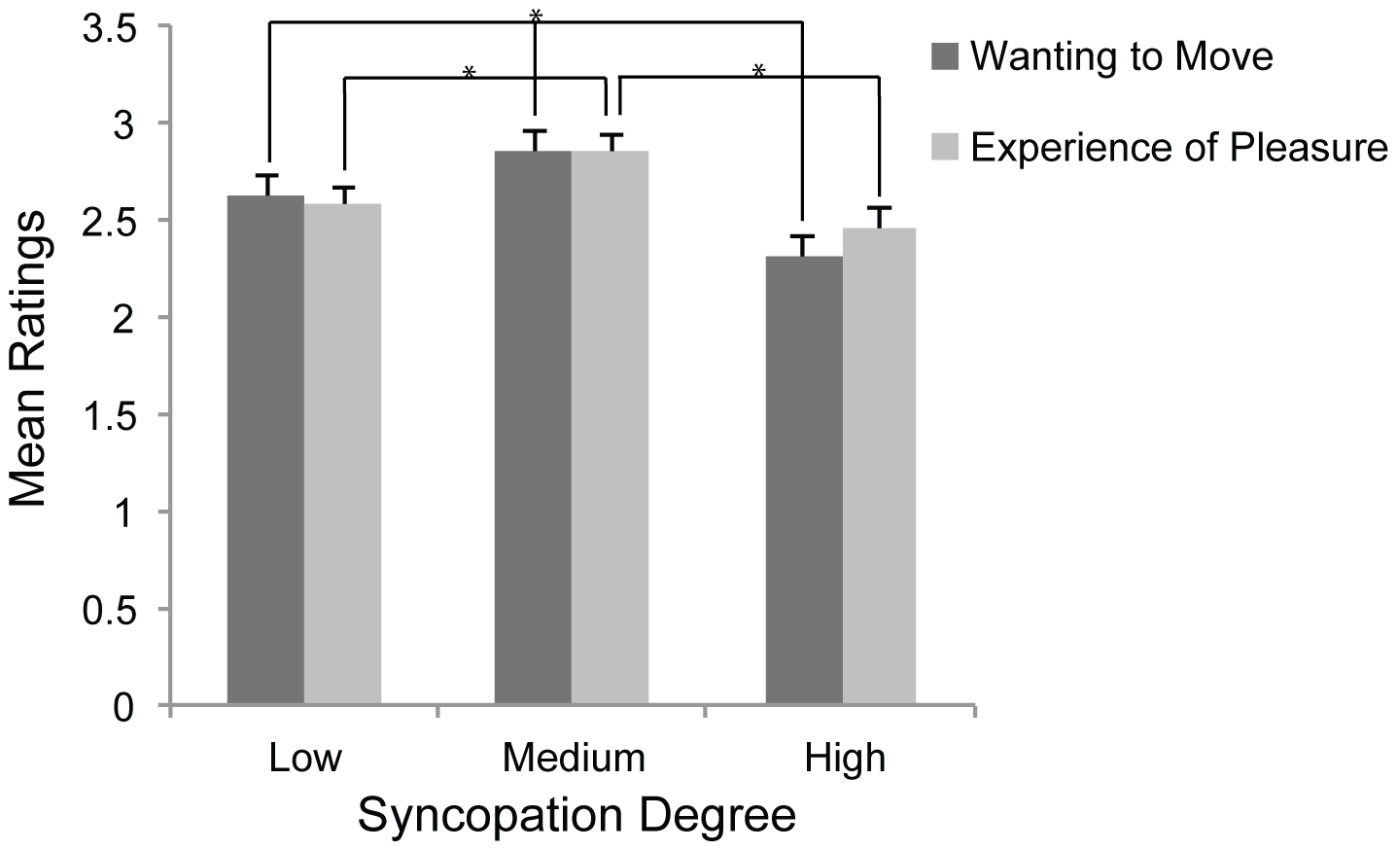
Effect of syncopation degree. Effect of 3-level parametric levels of syncopation degree – Low, Medium and High – on ratings of wanting to move and experience of pleasure. Error bars  =  standard error. *Alpha adjusted *p*<.005.

There was no main effect of rating question (*F*(1, 49)  = .35, *p* = .556, sphericity assumed) and a Pearson's correlation showed a significant strong correlation between wanting to move and experience of pleasure (*r* = .964, p<.001). A main effect was found for syncopation (*F*(1.62, 79.15)  = 15.73, *p*<.001, Greenhouse-Geisser corrected df), but there were no significant interactions between S and any between-subjects factors. There was, however, a significant interaction between S and rating question (*F*(1.79, 87.66)  = 6.823, *p* = .003, Greenhouse-Geisser corrected df). Paired t-tests (corrected for multiple comparisons; Table 5, Figure 4) showed that there were significant differences between all three levels for movement ratings, and that Medium syncopation was rated as eliciting the most desire to move, followed by Low and High, respectively. For pleasure ratings, however, Medium was rated higher than both Low and High, but there was no significant difference between Low and High. Despite this difference in the two rating questions, correction for multiple comparison yielded a nonsignificant contrast between High movement and High pleasure. There were neither any significant differences between wanting to move and feelings of pleasure for the Low or Medium categories.

**Table 5:** Paired contrasts for syncopation and rating question on ratings.

	Contrasts	Mean	SE	t	p
1	Low Movement vs Medium Movement	−.31	.05	−6.51	<.001*
2	Low Movement vs High Movement	.25	.07	3.41	.001*
3	Medium Movement vs High Movement	.55	.06	8.88	<.001*
4	Low Pleasure vs Medium Pleasure	−.32	.05	−6.07	<.001*
5	Low Pleasure vs High Pleasure	.07	.08	0.77	.441
6	Medium Pleasure vs High Pleasure	.39	.07	5.57	<.001*
7	Low Movement vs Low Pleasure	.05	.05	0.95	.348
8	Medium Movement vs Medium Pleasure	.03	.05	0.60	.552
9	High Movement vs High Pleasure	−.14	.06	−2.38	.020

Notes: Effect of 3-level parametric levels of syncopation – Low, Medium and High – on ratings of Movement  =  wanting to move, and Pleasure  =  experience of pleasure. Degrees of freedom  = 49, *Alpha adjusted *p*<.005.

There was a main effect of entropy (*F*(2, 98)  = 7.80, *p* = .001), but no interactions between JAE, rating questions or any between-subjects factors. Bonferroni-corrected post-hoc tests showed that High JAE (Mean  = 2.70, SE = .10) was rated significantly higher than Low JAE (Mean  = 2.51, SE  = .09) (*p* = .002), but that there were no significant differences between Medium (Mean  =  2.62, SE  = .09) and Low (*p* = .083), or Medium and High (*p* = .220).

In sum, enjoyment and frequency of dancing significantly affects ratings of wanting to move and pleasure. It was confirmed that medium degree of syncopation was the optimal level of syncopation with regards to ratings, but some differences were found in the exact shape of the inverted U-shape, depending on whether participants rated wanting to move or pleasure. Furthermore, there were suggestions of a positive linear relationship between joint audio entropy and ratings.

## Discussion

Using a web-based rating survey we found an inverted U-shaped relationship between degree of syncopation in drum-breaks and movement- and pleasure-ratings, indicating that intermediate degrees of syncopation elicit the most desire to move and pleasure in music associated with groove. As the syncopation in the drum-breaks increased, ratings increased accordingly, but only to an optimal point, after which a continued increase in syncopation caused decreasing movement desire and pleasure. Thus, the study shows that not just liking and preference [45], [48], but also motivation for overt action tendencies, such as sensorimotor synchronisation, is related to structural complexity in an inverted U-shaped way. In other words, Berlyne's theory of optimal perceptual stimulation in art [48] can be applied to models of affective engagements with music involving body-movement and dance. Syncopation predicted the inverted U-shaped relationship better than joint audio entropy, supporting previous evidence of syncopation outperforming entropy in modelling of perceptual complexity in rhythm [84]. Musical background affected ratings, in accordance with previous studies into contextual aspects of the inverted U-curve in music [46], [51], [52]. Ratings were amplified by people's experience with dancing, but were not significantly affected by musical training or familiarity with groove. Thus, our findings indicate that overt body-movement in dance influences the effects of syncopation on subjective experience of groove more robustly than peoples' previous experiences of playing music or listening to groove.

Although ratings of wanting to move and feelings of pleasure correlated strongly, confirming previous research [9], [10], [40], different levels of syncopation elicited wanting to move and feelings of pleasure differently: specifically, although drum-breaks with medium degrees of syncopation were rated highest for both movement and pleasure, low degrees of syncopation were rated higher than high degrees of syncopation for movement ratings only. While drum-breaks with too much syncopation may prevent successful entrainment and thus inhibit the desire to move, it may be that feelings of pleasure are still elicited that are unrelated to groove – for instance, high levels of syncopation may be associated with ‘free jazz’, in which irregular and unpredictable metre is common and aesthetically appropriate [97], [98]. Our findings suggest that drum-breaks with intermediate degrees of syncopation are more appropriate examples of musical groove, since they elicit the desire to move and feelings of pleasure equally, and to a greater extent than drum-breaks with either too little or too much syncopation. However, since there was no significant difference between the two rating questions at any level of syncopation (only a difference between levels *within* each rating question), these interpretations require further study before confident conclusions can be drawn.

Compared to syncopation, joint audio entropy was a poor predictor of the ratings collected in our study. Linear and quadratic fits between entropy and ratings could not be properly distinguished, and when considered alongside syncopation, it did not add any more explanatory power than already provided by the syncopation measure. However, a trend towards a positive linear relationship was found between entropy and ratings. Although our findings are in accordance with previous research showing improved performance of syncopation measures compared to entropy when modelling behavioural responses to rhythmic complexity [84], our results also suggest that, on its own, entropy is able to model some variability in ratings of wanting to move and experience of pleasure. The positive linear function for entropy suggests that listeners prefer grooves with high compared to low degrees of entropy as measured for the audio signal. It should be noted that there was a small but close-to-significant positive correlation between joint audio entropy and syncopation. In fact, for wanting to move, Low syncopation was rated significantly higher than High syncopation. In other words, when ignoring the Medium category, the relationship between syncopation and ratings was linear, just like for entropy. Thus, it could be that although medium degrees of syncopation optimise wanting to move and pleasure in response to groove, listeners prefer less complexity to more complexity, more generally.

Our results are of interest to researchers concerned with establishing the sparsely demonstrated link between entrainment and affect in music [10], [30], [40]. Although previous studies of groove have suggested that pleasure is involved, empirical evidence has been more consistent for sensorimotor synchronisation [7]–[10]. Here, we show that, in groove, the rhythms that make people want to move also elicit feelings of pleasure and we add to the theory that emotions are grounded in the body [1]–[4] by showing that in groove, desire for body-movement is pleasurable. Furthermore, our findings indicate that affective responses to rhythmic entrainment are optimised when the music involves an intermediate degree of syncopation. In other words, entrainment feels good when there is some structural resistance against the regular pulse in the musical material [99], [100]. This structural resistance could be the result of the violation of expectation that researchers often refer to when defining syncopation [44], [65], [79], [85], [101] and which is maximised at medium degrees of syncopation. With low degrees of syncopation, all or most metric expectations are confirmed, since there is little or no syncopation to violate them; and with high degrees of syncopation, there are only weak expectations to be violated, since the high degree of complexity disrupts metre perception and hence the generation of metric expectations. Medium degrees of syncopation, however, may provide just the right balance between sufficient rhythmic predictability for metre to be perceived and metrical expectations to occur, and sufficient complexity for those expectations to be violated and thus pleasure to be released [102].

A difficulty for an expectation-based account of the pleasure of groove is that the characteristically constant repetition of the syncopated rhythms should lead to decreasing rhythmic unexpectedness and decreasing pleasure [18]. An alternative is that the structural resistance provided by syncopation elicits a pleasurable desire to move because syncopation requires a certain degree of active participation on the part of the listener [11], [57], [103]. In dancing and foot-tapping to groove-based music, body movements are beat-directed and periodic [10], [36], so that sensorimotor synchronisation to syncopated rhythm becomes a corporeal enactment of metre. In this way, syncopation in music associated with groove could be seen as an *invitation* to the body to synchronise with the metre, the desire to move may be a *response* to this invitation and the pleasure a *result* of the fulfilled desire. Such a dynamic view of pleasure in groove adds to previous theories of pleasure cycles, both in biological reward [104], [105] and music [15], by suggesting that the body can play an active role in the anticipation and fulfilment of reward. Furthermore, we speculate that pleasure can occur at a more constant level, since body-movement in groove is continuously synchronised to the regular and repetitive beat, as opposed to directed towards one ‘chill’-inducing ‘peak’ structural moment.

The only category of musical background that affected ratings significantly was the extent to which participants liked to dance, and the frequency with which they danced to music. It may be that the type of active engagement that defines groove most consistently – namely dance and body-movement – is more closely related to the desire to move and feelings of pleasure than listeners' previous experience of listening to groove-based music and their formal musical training. It is interesting that our study only showed a close-to-significant effect of musical training, since musical expertise has been shown to affect sensorimotor synchronisation to and perceived stability of syncopated rhythms more generally [78], [93], [106] and movement induction to music associated with groove specifically [8]. It could be that when considering their affective experiences of music, listeners' propensity towards sensorimotor engagements with music is more influential than their performance skills, at least when listening to music associated with body-movement. However, since Keller and Schubert [44] found that syncopated melodies were more systematically related to affective rather than cognitive responses, it could also be that psychological effects of syncopation are better defined in terms of affect than cognitive skill. Nonetheless, since our study only considered subjective reports of wanting to move and feelings of pleasure, it remains to be determined whether more objective measures of pleasure differ for musicians and non-musicians and if musical training, groove familiarity and dance experience affect overt sensorimotor synchronisation.

In fact, the ways in which the ratings recorded in our study relate to overt body-movement in response to groove remain unknown more broadly. It could be that the relationship between syncopation and wanting to move changes when people are actually moving, and that the force of movement (which might be regarded as an index of the underlying desire to move) depends on rhythmic complexity in different ways. Furthermore, questions remain about the sensorimotor synchronisation to syncopated rhythm: in finger-tapping studies, degree of syncopation has been found to correlate linearly and negatively with finger-tapping accuracy [85], but no study has measured synchronisation in dance to syncopated rhythm. Although it might seem intuitively likely that the music that elicits the most desire to move also promotes the most successful synchronisation, there is no evidence to support this assumption.

Our study shows that across a wide range of nationalities, syncopation is related to wanting to move and pleasure in an inverted U-shaped way. However, the role of syncopation in music can differ according to culture, and thus culture-specific responses to syncopation may differ correspondingly [53], [54]. Our study leaves open the question whether culture affects the desire to move and experience of pleasure in response to syncopated drum-breaks, but shows that broadly, listeners prefer medium degrees of syncopation in groove.

Understanding what it is about music that motivates spontaneous affective and motor behaviour is of interest for music researchers, performers, educators and therapists. Our study is the first to demonstrate that in groove, pleasure and desire for body-movement are related to syncopation in an inverted U-shaped way, suggesting that Berlyne's theory of optimal perceptual stimulation in art [48] could be extended to include body-movement and dance. Since groove joins pleasure and sensorimotor synchronisation [10], both thought to promote adaptive functioning [16], [26]–[29], the study of groove furthers our knowledge about musical behaviour more broadly, a behaviour that remains uniquely human and culturally ubiquitous.

## Supporting Information

Figure S1 Notational transcripts and audio descriptor values.
(TIF)

Figure S2 Notational transcripts and audio descriptor values.
(TIF)

Figure S3 Notational transcripts and audio descriptor values.
(TIF)

Figure S4 Notational transcripts and audio descriptor values.
(TIF)

Figure S5 Model of metric salience.
(TIF)

Figure S6 Drum-break of ‘Lifetime Monologue’ by Lou Rawls.
(TIF)

Figure S7 Drum-breaks of ‘Impeach the President’ by Honeydrippers and ‘Actual Proof’ by Herbie Hancock.
(TIF)

Figure S8 Demographics questionnaire.
(TIF)

Figure S9 Rating survey.
(TIF)

Table S1 Descriptive statistics for three-level categorisation of syncopation.
(DOCX)

Text S1 Participant between-subjects categorization.
(DOCX)

Text S2 Index of syncopation.
(DOCX)

Text S3 Joint audio entropy.
(DOCX)

Text S4 Validation of syncopation index.
(DOCX)

Text S5 Controlling for ‘unusualness’ in experimenter-composed drum-breaks.
(DOCX)

Text S6 Effects of musical background on parabola vertex.
(DOCX)

## References

[1] Niedenthal PM, Barsalou LW, Winkielman P, Krauth-Gruber S, Ric F (2005) Embodiment in Attitudes, Social Perception, and Emotion. Personality and Social Psychology Review 9: 184–211. 16083360 10.1207/s15327957pspr0903_1

[2] Barrett LF, Lindquist K (2008) The embodiment of emotion. In: Semin GR, Smith ER, editors. Embodied grounding. Cambridge: Cambridge University Press.

[3] Niedenthal PM (2007) Embodying Emotion. Science 316: 1002–1005. 17510358 10.1126/science.1136930

[4] Winkielman P, Niedenthal PM, Oberman L (2008) The embodied emotional mind. In: Semin GR, Smith ER, editors. Embodied grounding Social, cognitive, affective and neuroscientific approaches. Cambridge: Cambrideg University Press. pp. 263–288.

[5] Wilson M (2002) Six views of embodied cognition. Psychonomic Bulletin & Review 9: 625–636. 12613670 10.3758/bf03196322

[6] Semin GR, Smith ER (2008) Embodied grounding: social, cognitive, affective, and neuroscientific approaches. Cambridge: Cambridge University Press.

[7] Madison G (2006) Experiencing groove induced by music: Consistency and phenomenology. Music Perception 24: 201–208.

[8] Stupacher J, Hove MJ, Novembre G, Schütz-Bosbach S, Keller PE (2013) Musical groove modulates motor cortex excitability: A TMS investigation. Brain and Cognition 82: 127–136. 23660433 10.1016/j.bandc.2013.03.003

[9] Madison G, Gouyon F, Ullén F, Hörnström K (2011) Modeling the tendency for music to induce movement in humans: First correlations with low-level audio descriptors across music genres. Journal of Experimental Psychology: Human Perception and Performance 37: 1578–1594. 21728462 10.1037/a0024323

[10] Janata P, Tomic ST, Haberman JM (2012) Sensorimotor coupling in music and the psychology of the groove. Journal of Experimental Psychology: General 141: 54–75. 21767048 10.1037/a0024208

[11] Danielsen A (2006) Presense and pleasure. The funk grooves of James Brown and Parliament. Middletown, Connecticut: Wesleyan University Press.

[12] Greenwald J (2002) Hip-hop drumming: The rhyme may define, but the groove makes you move. Black Music Research Journal 22: 259–271.

[13] Gioia T (2011) The history of jazz: Oxford University Press.

[14] Butler MJ (2006) Unlocking the groove: Rhythm, meter, and musical design in electronic dance music. Bloomington: Indiana University Press.

[15] Gebauer L, Kringelbach ML, Vuust P (2012) Ever-changing cycles of musical pleasure: The role of dopamine and anticipation. Psychomusicology 22: 152–167.

[16] Vuust P, Kringelbach ML (2010) The pleasure of music. In: Kringelbach ML, Berridge KC, editors. Pleasures of the brain. New York: Oxford University Press. pp. 255–269.

[17] Vuust P, Frith CD (2008) Anticipation is the key to understanding music and the effects of music on emotion. Behavioral and Brain Sciences 31: 599–600.

[18] Huron D (2006) Sweet anticipation: Music and the psychology of expectation. Cambridge, MA: The MIT Press. Sweet anticipation: Music and the psychology of expectation. xii, 462 p.

[19] Meyer LB (1956) Emotion and meaning in music. Chicago and London: University of Chicago Press.

[20] Gomez P, Danuser B (2007) Relationships between musical structure and psychophysiological measures of emtoion. Emotion 7: 377–387. 17516815 10.1037/1528-3542.7.2.377

[21] Grewe O, Nagel F, Kopiez R, Altenmuller E (2007) Listening to Music as a Re-Creative Process: Physiological, Psychological, and Psychoacoustical Correlates of Chills and Strong Emotions. Music Perception 24: 297–314.

[22] Grewe O, Kopiez R, Altenmuller E (2009) The chill parameter: Goose bumps and shivers as promising measures in emotion research. Music Perception 27: 61–74.

[23] Guhn M, Hamm A, Zentner M (2007) Physiological and musico-acoustic correlates of the chill response. Music Perception 24: 473–483.

[24] Krumhansl CL (1997) An exploratory study of musical emotions and psychophysiology. Canadian Journal of Experimental Psychology/Revue canadienne de psychologie expérimentale 51: 336–353. 9606949 10.1037/1196-1961.51.4.336

[25] Ritossa DA, Rickard NS (2004) The relative utility of “pleasentness and liking” dimensions in predicting the emotions expressed by music. Psychology of Music 32: 5–22.

[26] Blood AJ, Zatorre RJ (2001) Intensely pleasurable responses to music correlate with activity in brain region implicated with reward and emotion. Proceedings of the National Academy of Sciences 98: 11818–11823. 10.1073/pnas.191355898 PMC58814 11573015

[27] Salimpoor VN, Benovoy M, Larcher K, Dagher A, Zatorre RJ (2011) Anatomically distinct dopamine release during anticipation and experience of peak emotion to music. Nature Neuroscience 14: 257–262. 21217764 10.1038/nn.2726

[28] Bispham J (2006) Rhythm in music: What is it? Who has it? And why? Music Perception 24: 125–134.

[29] Merker B, Madison G, Eckerdal P (2009) On the role and origin of isochrony in human rhythmic entrainment. Cortex 45: 4–17. 19046745 10.1016/j.cortex.2008.06.011

[30] Zentner M, Eerola T (2010) Rhythmic engagement with music in infancy. Proceedings of the National Academy of Sciences 107: 5768–5773. 10.1073/pnas.1000121107 PMC2851927 20231438

[31] Clayton M, Sager R, Will U (2004) In time with the music: The concept of entrainment and its significance for ethnomusicology. European Meetings in Ethnomusicology II (ESEM counterpoint 1): 3–75.

[32] Large EW, Jones MR (1999) The dynamics of attending: How people track time-varying events. Psychological Review 106: 119–159.

[33] Phillips-Silver J, Aktipis AC, Bryant G (2010) The ecology of entrainment: Foundations of coordinated rhythmic movement. Music Perception 28: 3–14. 21776183 10.1525/mp.2010.28.1.3 PMC3137907

[34] Large EW, Kolen JF (1994) Resonance and the perception of musical meter. Connection Science 6: 177–208.

[35] Jones MR (2009) Musical time. In: Hallam S, Cross I, Thaut M, editors. The Oxford handbook of music psychology. New York: Oxford University Press. pp. 81–92.

[36] Toiviainen P, Luck G, Thompson MR (2010) Embodied meter: Hierarchical eigenmodes in music-induced movement. Music Perception 28: 59–70.

[37] Fairhurst MT, Janata P, Keller PE (2012) Being and feeling in sync with an adaptive virtual partner: Brain mechanisms underlying dynamic cooperativity. Cerebral Cortex 67: 313–321. 10.1093/cercor/bhs243 22892422

[38] Keller PE (2008) Joint action in music performance. In: Morganti F, Carassa A, Riva G, editors. Enacting intersubjectivity: A cognitive and social perspective to the study of interactions. Amterdam: IOS Press. pp. 205–221.

[39] Repp B, Keller PE (2008) Sensorimotor synchronisation with adaptively timed sequences. Human Movement Science 27: 423–456. 18405989 10.1016/j.humov.2008.02.016

[40] Trost W, Vuilleumier P (2013) Rhythmic entrainment as a mechanism for emotion induction by music: A neurophysiological perspective. In: Cochrane T, Fantini B, Scherer KR, editors. The emotional power of music: multidisciplinary perspectives on musical arousal, expression, and social control. New York: Oxford University Press. pp. 213–225.

[41] McGuiness A, Overy K (2011) Music, consciousness and the brain. In: Clarke D, Clarke EF, editors. Music and consciousness: Philosophical, psychological, and cultural perspectives. New York: Oxford University Press. pp. 245–271.

[42] Overy K, Molnar-Szakacs I (2009) Being together in time: Musical experience and the mirror neuron system. Music Perception 26: 489–504.

[43] Sloboda JA (1991) Music structure and emotional response: Some empirical findings. Psychology of Music 19: 110–120.

[44] Keller PE, Schubert E (2011) Cognitive and affective judgements of syncopated musical themes. Advances in Cognitive Psychology 7: 142–156. 22253676 10.2478/v10053-008-0094-0 PMC3259101

[45] North AC, Hargreaves DJ (1995) Subjective complexity, familiarity, and liking for popular music. Psychomusicology 14: 77–93.

[46] North AC, Hargreaves DJ (1997) Experimental aesthetics and everyday music listening. In: Hargreaves DJ, North AC, editors. The social psychology of music. Oxford: Oxford University Press.

[47] North AC, Hargreaves DJ (1998) Complexity, propotypicality, familiarity, and the perception of musical quality. Psychomusicology 17: 77–80.

[48] Berlyne DE (1971) Aesthetics and psychobiology. East Norwalk, CT: Appleton-Century-Crofts.

[49] Wundt W (1874) Grundzuge der physiologischen psychologie. Leipzig: Englemann.

[50] Heyduk RG (1975) Rated preference for musical compositions as it relates to complexity and exposure frequency. Perception and Psychophysics 17: 84–91.

[51] McNamara L, Ballard ME (1999) Resting arousal, sensation seeking, and music preference. Genetic, Social, and General Psychology Monographs 125: 229–250.

[52] Orr MG, Ohlsson S (2005) Relationship between complexity and liking as a function of expertise. Music Perception 22: 583–611.

[53] Hannon EE, Soley G, Ullal S (2012) Familiarity overrides complexity in rhythm perception: A cross-cultural comparison of American and Turkish listeners. Journal of Experimental Psychology: Human Perception and Performance 38: 543–548. 22352419 10.1037/a0027225

[54] Roncaglia-Denissen MP, Schmidt-Kassow M, Heine A, Vuust P, Kotz SA (2013) Enhanced musical rhythmic perception in Turkish early and late learners of German. Frontiers in psychology 4: 645–653. 24065946 10.3389/fpsyg.2013.00645 PMC3778315

[55] Waadeland CH (2001) “It don't mean a thing if it ain't got that swing” – Simulating expressive timing by modulated movements. Journal of New Music Research 30: 23–37.

[56] Iyer V (2002) Embodied mind, situated cognition, and expressive microtiming in African-American music. Music Perception 19: 387–414.

[57] Keil C, Feld S (1994) Music grooves: Essays and dialogues. Chicago, London: University of Chicago Press.

[58] Davies M, Madison G, Silva P, Gouyon F (2013) The effect of microtiming deviations on the perception of groove in short rhythms. Music Perception: An Interdisciplinary Journal 30: 497–510.

[59] Clarke EF (1989) The perception of expressive timing in music. Psychological Research 51: 2–9. 2756071 10.1007/BF00309269

[60] Repp BH (1992) Diversity and commonality in music performance: An analysis of timing microstructure in Schumann's “Träumerei”. The Journal of the Acoustical Society of America 92: 2546. 1479119 10.1121/1.404425

[61] Johansson M (2010) The concept of rhythmic tolerance: Examining flexible grooves in Scandinavian folk fiddling. In: Danielsen A, editor. Musical rhythm in the age of digital reproduction. Farnham: Ashgate. pp. 69–84.

[62] Madison G (2000) Properties of expressive variability patterns in music performances. Journal of New Music Research 29: 335–356.

[63] Danielsen A (2010) Here, there and everywhere: three accounts of pulse in D'Angelo's ‘Left and Right’. In: Danielsen A, editor. Musical rhythm in the age of digital reproduction. Farnham: Ashgate. pp. 19–38.

[64] Carlsen K, Witek MAG (2010) Simultaneous rhythmic events with different schematic affiliations: Microtiming and dynamic attending in two comtemporary R&B grooves. In: Danielsen A, editor. Musical rhythm in the age of digital reproduction. Farnham: Ashgate. pp. 51–68.

[65] Longuet-Higgins HC, Lee C (1984) The rhythmic interpretation of monophonic music. Music Perception 1: 424–440.

[66] Temperley D (1999) Syncopation in rock: a perceptual perspective. Popular Music 18: 19–40.

[67] Zbikowski L (2004) Modelling the groove: Conceptual structure and popular music. Journal of the Royal Musical Association 129: 272–297.

[68] Pressing J (2002) Black Atlantic rhythm: Its computational and transcultural foundations. Music Perception 19: 285–310.

[69] Bengtsson SL, Ullén F, Henrik Ehrsson H, Hashimoto T, Kito T, et al (2009) Listening to rhythms activates motor and premotor cortices. Cortex 45: 62–71. 19041965 10.1016/j.cortex.2008.07.002

[70] Chapin HL, Zanto T, Jantzen KJ, Kelso SJA, Steinberg F, et al (2010) Neural responses to complex auditory rhythms: The role of attending. Frontiers in Psychology 1: 1–18. 21833279 10.3389/fpsyg.2010.00224 PMC3153829

[71] Chen JL, Penhune VB, Zatorre RJ (2006) Interactions between auditory and dorsal premotor cortex during synchronization to musical rhythms. Neuroimage 32: 1771–1781. 16777432 10.1016/j.neuroimage.2006.04.207

[72] Chen JL, Penhune VB, Zatorre RJ (2008) Listening to musical rhythms recruits motor regions of the brain Cerebral Cortex. 18: 2844–2854. 10.1093/cercor/bhn042 18388350

[73] Grahn JA, Brett M (2007) Rhythm and beat perception in motor areas of the brain. Journal of Cognitive Neuroscience 19: 893–906. 17488212 10.1162/jocn.2007.19.5.893

[74] Grahn JA, Rowe JB (2009) Feeling the beat: premotor and striatal interactions in musicians and nonmusicians during beat perception. The Journal of Neuroscience 29: 7540–7548. 19515922 10.1523/JNEUROSCI.2018-08.2009 PMC2702750

[75] Grahn JA, Rowe JB (2012) Finding and feeling the musical beat: Striatal dissociations between detection and prediction of regularity. Cerebral Cortex 23: 913–921. 22499797 10.1093/cercor/bhs083 PMC3593578

[76] Nozaradan S, Peretz I, Missal M, Mouraux A (2011) Tagging the neuronal entrainment to beat and meter. Journal of Neuroscience 31: 10234–10240. 21753000 10.1523/JNEUROSCI.0411-11.2011 PMC6623069

[77] Honing H (2012) Without it no music: beat induction as a fundamental musical trait. Annals of the New York Academy of Sciences 1252: 85–91. 22524344 10.1111/j.1749-6632.2011.06402.x

[78] Palmer C, Krumhansl CL (1990) Mental representation for musical meter. Journal of Experimental Psychology: Human Perception and Performance 16: 728–741. 2148588 10.1037//0096-1523.16.4.728

[79] Ladinig O, Honing H, Haden G, Winkler I (2009) Probing attentive and preattentive emergent meter in adult listeners without extensive musical training. Music Perception 26: 377–386.

[80] Povel D-J, Essens P (1985) Perception of temporal patterns. Music Perception 2: 411–440. 10.3758/bf03207132 3991313

[81] Shmulevich I, Povel D-J (2000) Complexity measures of musical rhythms. In: Desain P, Windsor L, editors. Rhythm perception and production. Lisse, NL.: Swets & Zeitlinger. pp. 239–244.

[82] Toussaint GT (2002) A mathematical analysis of African, Brazilian, and Cuban clave rhythms. BRIDGES: Mathematical Connections in Art, Music and Science: 23–27.

[83] Gomez F, Melvin A, Rappaport D, Toussaint GT (2005) Mathematical measures of syncopation. BRIDGES: Mathematical Connections in Art, Music and Science: 73–84.

[84] Thul E, Toussaint GT (2008) Rhythm complexity measures: A comparison of mathematical models of human perception and performance. Proceedings of the Ninth International Symposium on Music Information Retrieval (ISMIR): 663–668.

[85] Fitch WT, Rosenfeld AJ (2007) Perception and production of syncopated rhythms. Music Perception 25: 43–58.

[86] Smith J, Honing H (2006) Evaluation and extending computational models of rhythmic syncopation in music. Proceedings of the international computer music conference, New Orleans, LA.

[87] Margulis EH, Beatty AP (2008) Musical style, psychoaesthetics, and prospects for entropy as an analytical tool. Computer Music Journal 32: 64–78.

[88] Temperley D (2010) Modeling common-practice rhythm. Music Perception 27: 355–376.

[89] Large EW (2000) On synchronizing movements to music. Human Movement Science 19: 527–566.

[90] Mayville JM, Fuchs A, Ding M, Cheyne D, Deecke L, et al (2001) Event-related changes in neuromagnetic activity associated with sycnopation and synchronization timing tasks. Human Brain Mapping 14: 65–80. 11500991 10.1002/hbm.1042 PMC6872034

[91] Snyder JS, Krumhansl CL (2001) Tapping to ragtime: Cues to pulse finding. Music Perception 18: 455–489.

[92] Thaut M, Kenyon GP (2003) Rapid motor adaptations to subliminal frequency shifts during syncopated rhythmic sensorimotor synchronization. Human Movement Science 22: 321–338. 12967761 10.1016/s0167-9457(03)00048-4

[93] Ladinig O (2009) Temporal expectations and their violations. [Doctoral thesis]: University of Amsterdam.

[94] Winkler I, Haden G, Ladinig O, Sziller I, Honing H (2009) Newborn infants detect the beat in music. Proceedings of the National Academy of Sciences of the United States of America 106: 1–4. 10.1073/pnas.0809035106 PMC2631079 19171894

[95] MacKay DJ (2003) Information theory, inference and learning algorithms. Cambridge: Cambridge university press.

[96] Temperley D (2007) Music and probability. Campridge, MA: MIT Press.

[97] Vuust P, Roepstorff A (2008) Listen up! Polyrhythms in Brain and Music. Cognitive Semiotics 2008: 134–158.

[98] Leino S, Brattico E, Tervaniemi M, Vuust P (2007) Representation of harmony rules in the human brain: further evidence from event-related potentials. Brain Research 1142: 169–177. 17300763 10.1016/j.brainres.2007.01.049

[99] Vuust P, Roepstorff A, Wallentin M, Mouridsen K, Østergaard L (2006) It don't mean a thing…: Keeping the rhythm during polyrhythmic tension, activates language areas (BA47). Neuroimage 31: 832–841. 16516496 10.1016/j.neuroimage.2005.12.037

[100] Vuust P, Wallentin M, Mouridsen K, Østergaard L, Roepstorff A (2011) Tapping polyrhythms in music activates language areas. Neuroscience Letters 494: 211–216. 21397659 10.1016/j.neulet.2011.03.015

[101] Vuust P, Ostergaard L, Pallesen KJ, Bailey C, Roepstorff A (2009) Predictive coding of music-brain responses to rhythmic incongruity. Cortex 45: 80–92. 19054506 10.1016/j.cortex.2008.05.014

[102] Vuust P, Frith CD (2008) Anticipation is the key to understanding music and the effects of music on emotion. Behavioral and Brain Sciences 31: 599–600.

[103] Witek MAG (2013) ‘… and I feel good!’ The relationship between body-movement, pleasure and groove in music [Doctoral thesis]: University of Oxford.

[104] Kringelbach ML, Stein A, van Hartevelt TJ (2012) The functional human neuroanatomy of food pleasure cycles. Physiology & behavior 106: 307–316. 22487544 10.1016/j.physbeh.2012.03.023

[105] Georgiadis JK, Kringelbach ML (2012) The human sexual response cycle: Brain imaging evidence linking sex to other pleasures. Progress in Neurobiology 98: 49–81. 22609047 10.1016/j.pneurobio.2012.05.004

[106] Vuust P, Pallesen KJ, Bailey C, van Zuijen TL, Gjedde A, et al (2005) To musicians, the message is in the meter: pre-attentive neuronal responses to incongruent rhythm are left-lateralized in musicians. Neuroimage 24: 560–564. 15627598 10.1016/j.neuroimage.2004.08.039

