# Correction: Syncopation, Body-Movement and Pleasure in Groove Music

**DOI:** 10.1371/journal.pone.0139409

**Published:** 2015-09-24

**Authors:** Maria A. G. Witek, Eric F. Clarke, Mikkel Wallentin, Morten L. Kringelbach, Peter Vuust

There are errors in the stimulus indexing and notational transcripts found after further analysis in Supporting Information S1–S4 Figs, S7 Fig, S1 Table, and S2 Text. The authors declare that the errors do not affect the outcome, interpretations or significance of the study findings, and have provided corrected files here.

## Supporting Information

S1 Fig Notational transcripts and audio descriptor values. (PDF)

S2 Fig Notational transcripts and audio descriptor values. (PDF)

S3 Fig Notational transcripts and audio descriptor values. (PDF)

S4 Fig Notational transcripts and audio descriptor values. (PDF)

S7 Fig Drum-breaks of ‘Impeach the President’ by Honeydrippers and ‘Actual Proof’ by Herbie Hancock. (PDF)

S1 Table Descriptive statistics for three-level categorisation of syncopation. (DOCX)

S2 Text Index of syncopation. (DOCX)
