# Supplementary figures and images for: Syncopation, Body-Movement and Pleasure in Groove Music
Source: PLoS One. 2014 Apr 16;9(4):e94446. doi: 10.1371/journal.pone.0094446 (PMC3989225; doi:10.1371/journal.pone.0094446)

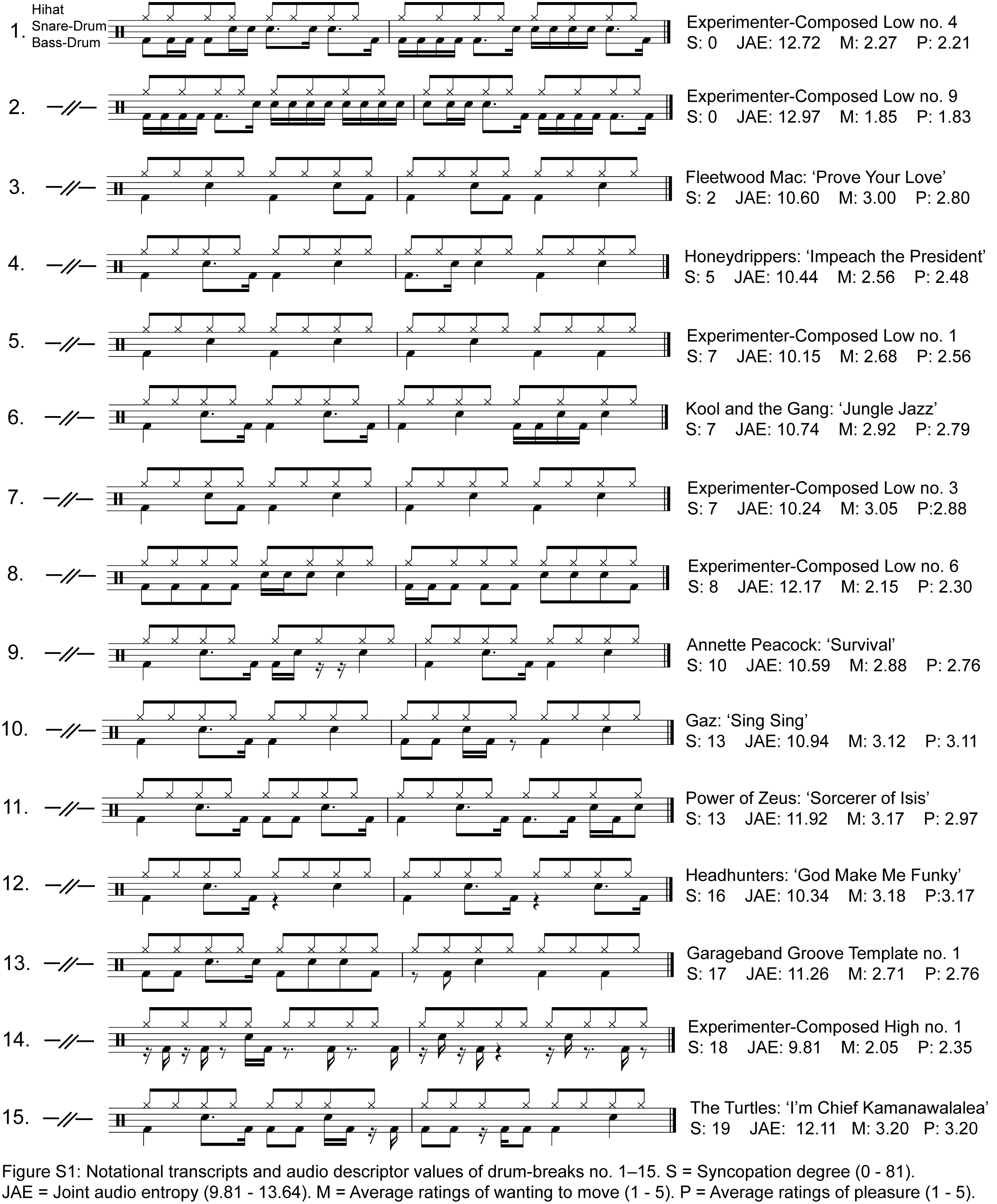

Supplement: Figure S1 — Notational transcripts and audio descriptor values. (TIF) [file pone.0094446.s001.tif]

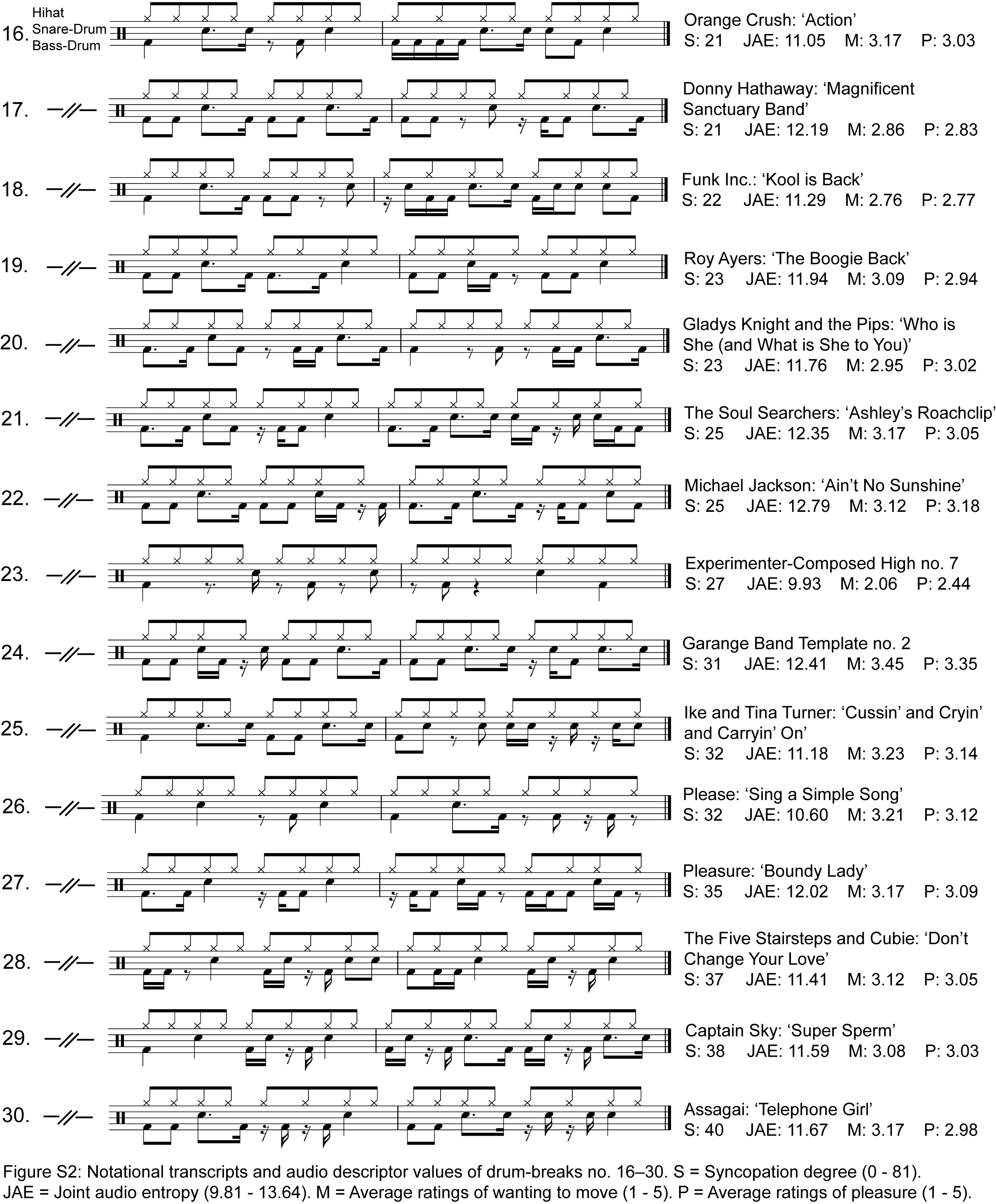

Supplement: Figure S2 — Notational transcripts and audio descriptor values. (TIF) [file pone.0094446.s002.tif]

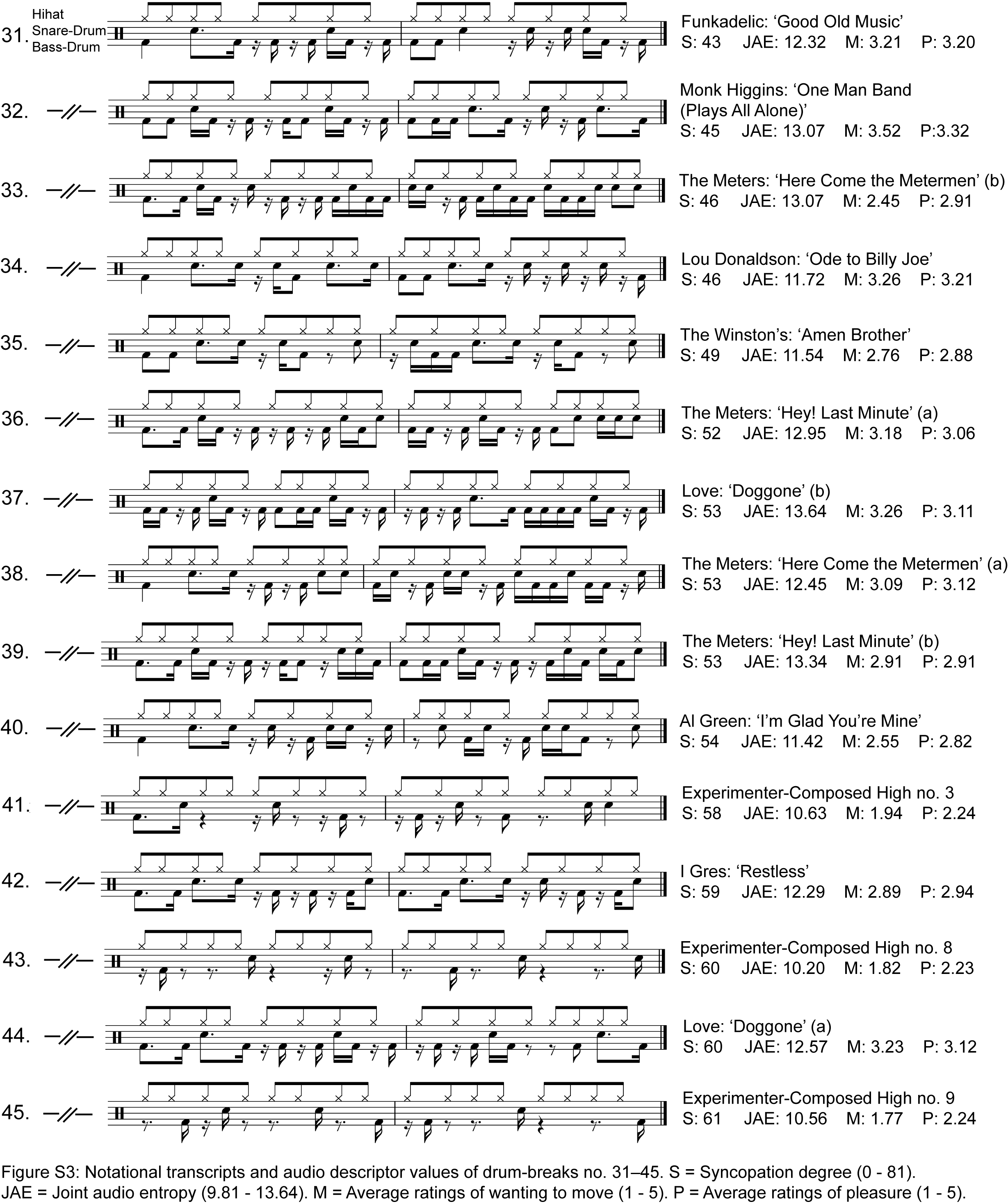

Supplement: Figure S3 — Notational transcripts and audio descriptor values. (TIF) [file pone.0094446.s003.tif]

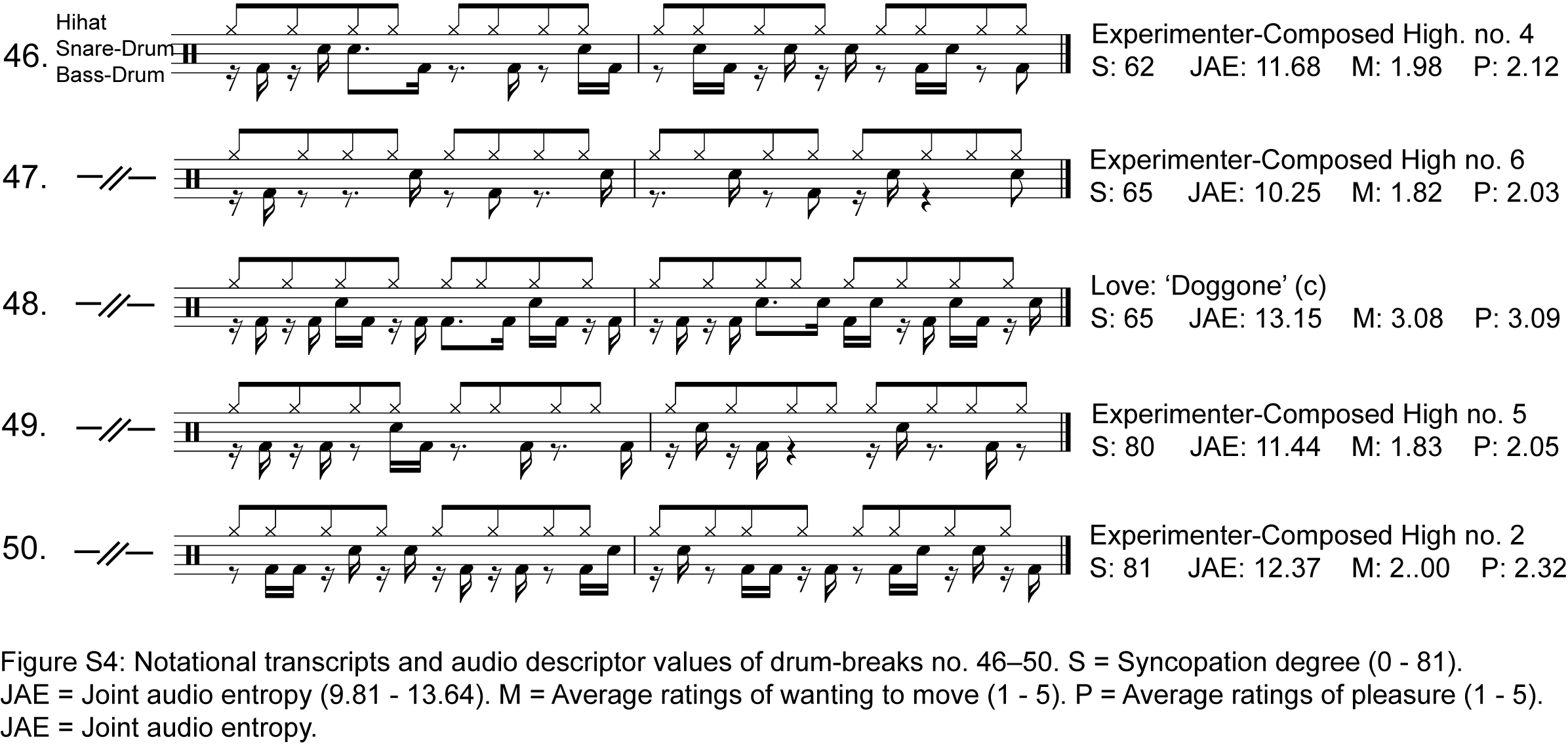

Supplement: Figure S4 — Notational transcripts and audio descriptor values. (TIF) [file pone.0094446.s004.tif]

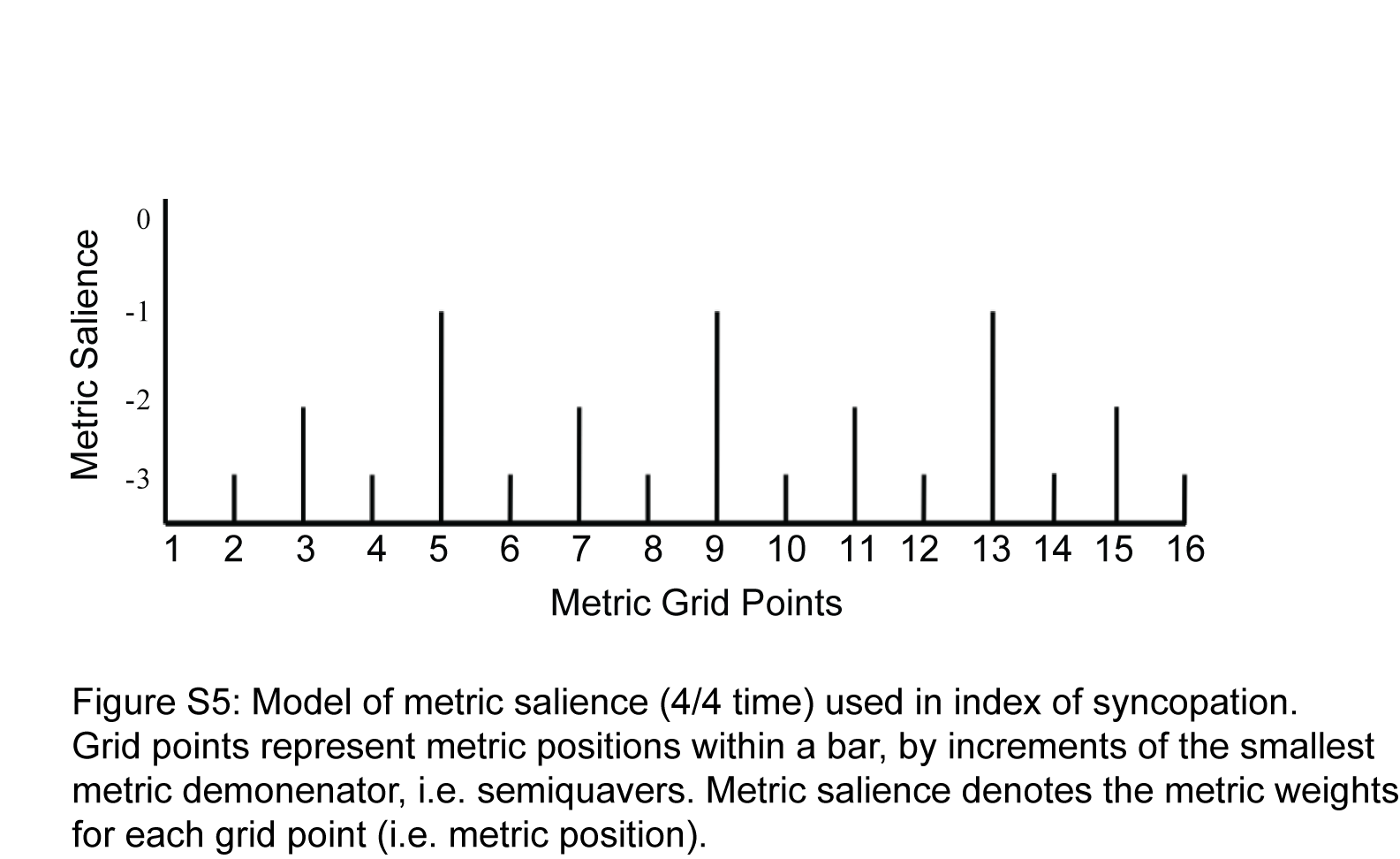

Supplement: Figure S5 — Model of metric salience. (TIF) [file pone.0094446.s005.tif]

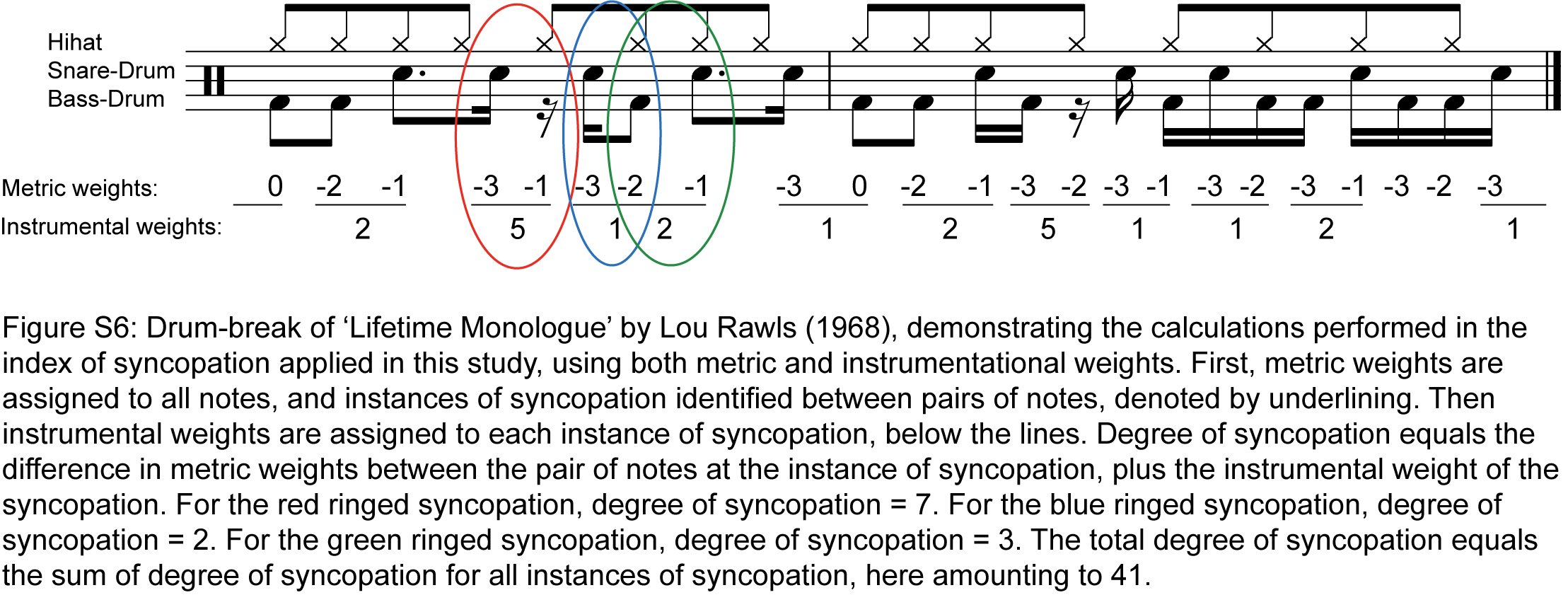

Supplement: Figure S6 — Drum-break of ‘Lifetime Monologue’ by Lou Rawls. (TIF) [file pone.0094446.s006.tif]

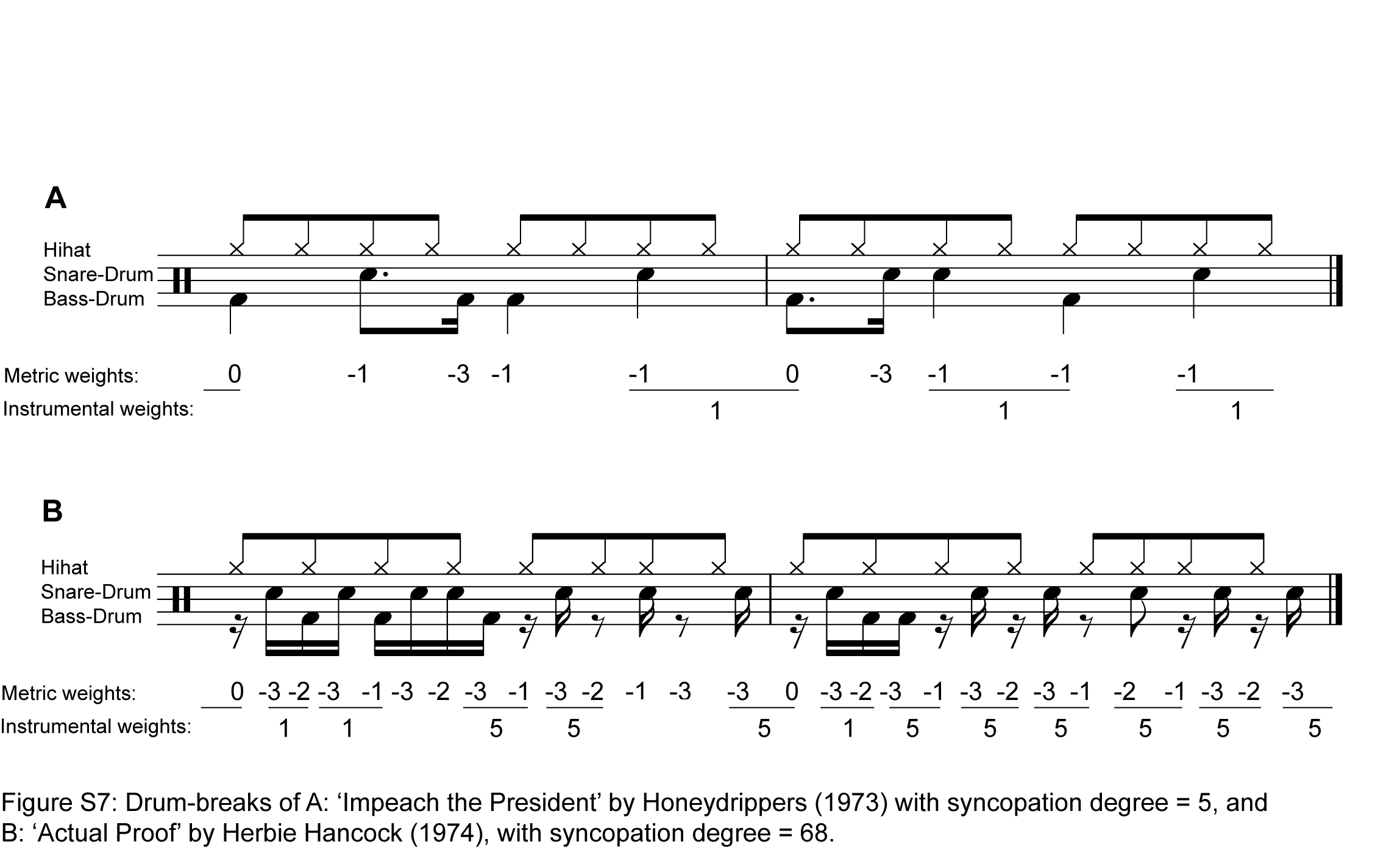

Supplement: Figure S7 — Drum-breaks of ‘Impeach the President’ by Honeydrippers and ‘Actual Proof’ by Herbie Hancock. (TIF) [file pone.0094446.s007.tif]

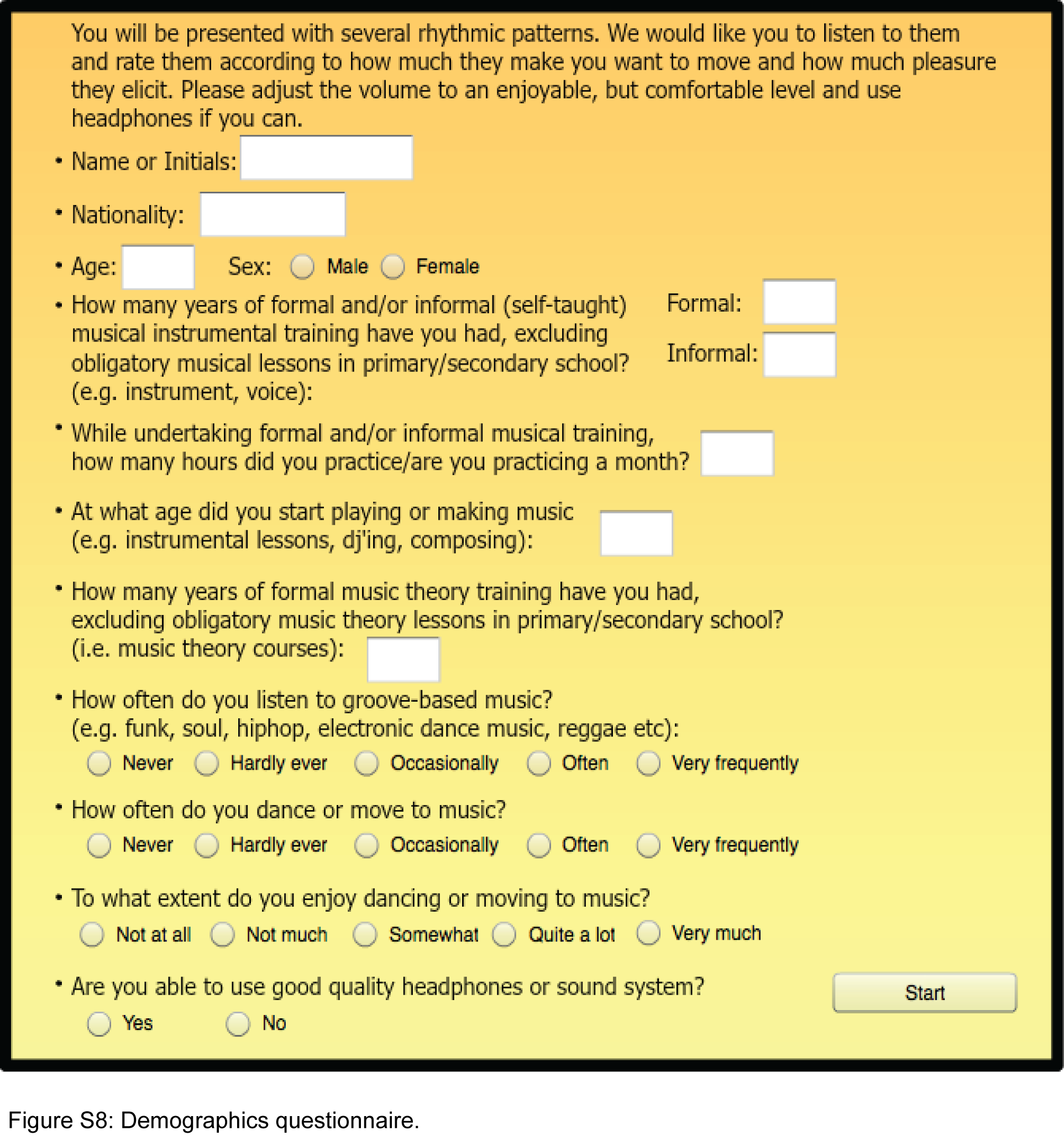

Supplement: Figure S8 — Demographics questionnaire. (TIF) [file pone.0094446.s008.tif]

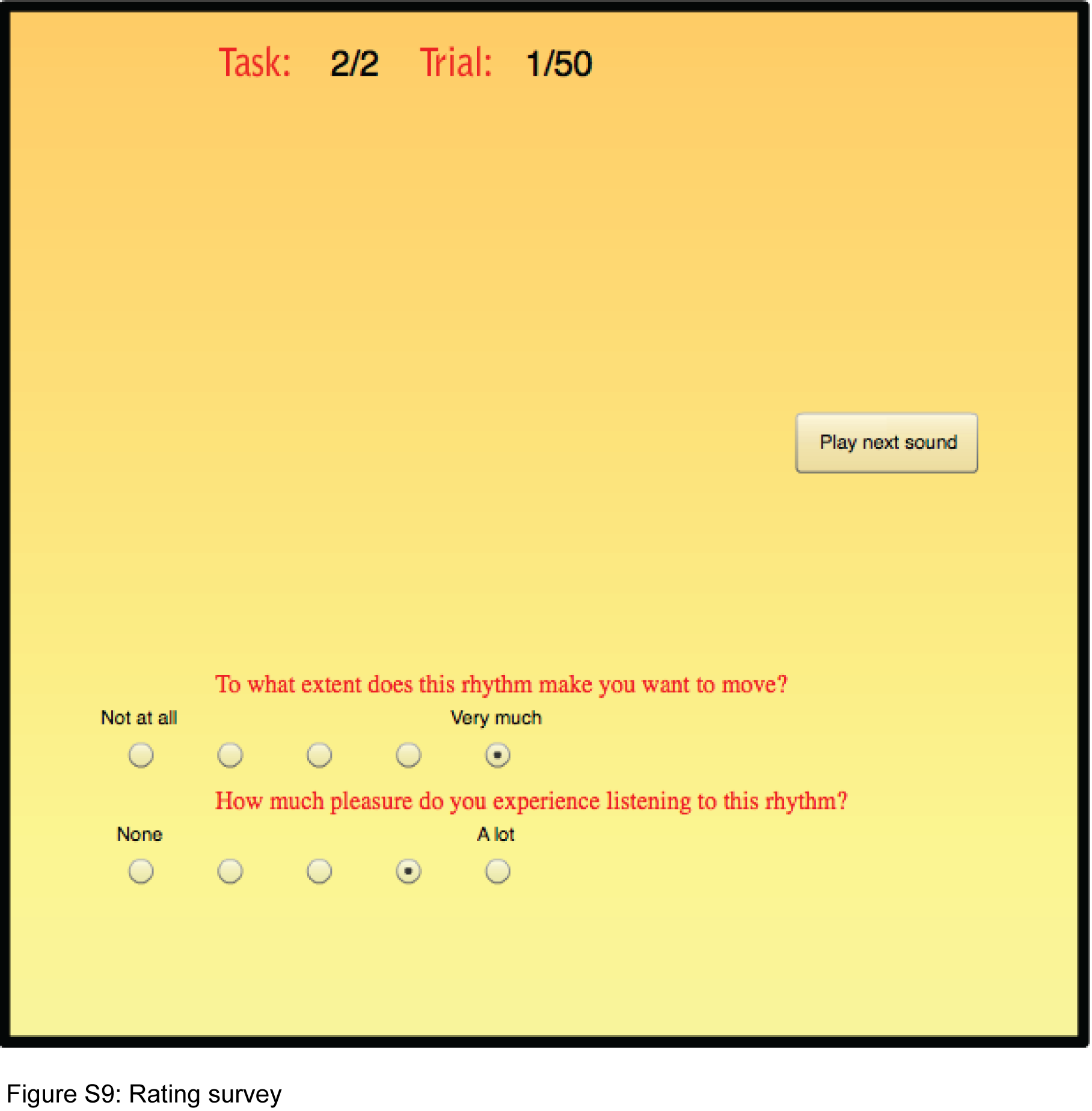

Supplement: Figure S9 — Rating survey. (TIF) [file pone.0094446.s009.tif]
