# Supplementary material for: Syncopation, Body-Movement and Pleasure in Groove Music
Source: PLoS One. 2014 Apr 16;9(4):e94446. doi: 10.1371/journal.pone.0094446 (PMC3989225; doi:10.1371/journal.pone.0094446)
Supplement: Table S1 — Descriptive statistics for three-level categorisation of syncopation. (DOCX) [file pone.0094446.s010.docx]

Supporting information Table S1

**Table S1. Descriptive statistics for three-level categorisation of syncopation**

| **Predictors** | | **N (stimuli)** | **Mean index value** | **Minimum** | **Maximum** |
| --- | --- | --- | --- | --- | --- |
| Syncopation | Low | 17 | 10.82 | 0 | 21 |
|  | Medium | 17 | 33.53 | 22 | 46 |
|  | High | 16 | 60.31 | 49 | 81 |
| Joint Audio Entropy | Low | 17 | 10.48 | 9.81 | 11.18 |
|  | Medium | 17 | 11.71 | 11.26 | 12.29 |
|  | High | 16 | 12.78 | 12.31 | 13.64 |
