# Supplementary material for: Syncopation, Body-Movement and Pleasure in Groove Music
Source: PLoS One. 2014 Apr 16;9(4):e94446. doi: 10.1371/journal.pone.0094446 (PMC3989225; doi:10.1371/journal.pone.0094446)
Supplement: Text S1 — Participant between-subjects categorization. (DOCX) [file pone.0094446.s011.docx]

Supporting information Text S1

*Participant between-subjects categorisation.*

Participants’ background in music, dance and groove was categorised in the following way: those with more than 8 years of formal and/or informal (self-taught) musical training were categorised as ‘musicians’, while those with less than 4 years of training were categorised as ‘non-musicians’, following criteria from previous research [[1](#_ENREF_1),[2](#_ENREF_2)]. Nine participants fell outside of these categories and were excluded, making the total number of participant for analyses involving between-subjects effects 57. The information regarding familiarity with groove and dancing experience were recorded on 5-point Likert scales. Those who rated the frequency with which they listened to groove-based music as 4 or 5 on the Likert scale (‘often’ or ‘very frequently’) were categorised as ‘groove-listeners’, while those who rated between 1 and 3 (‘never’, ‘hardly ever’ or ‘occasionally’) were categorised as ‘non-groove-listeners’. Those who rated 4 or 5 for the frequency with which they danced to music (‘often’ or ‘very frequently’), and for the extent to which they liked dancing to music (‘quite a lot’ or ‘very much’), were categorised as ‘dancers’. Those who rated between 1 and 3 for frequency of dancing (‘never’, ‘hardly ever’ or ‘occasionally’), and dance liking (‘not at all’, ‘not much’ or ‘somewhat’), were categorised as ‘non-dancers’. Pearson’s *r* test showed that there were no correlations between groove familiarity and dance experience (*r* = .209, *p* = .469).

References

1. Ladinig O (2009) Temporal expectations and their violations. [Doctoral thesis]: University of Amsterdam.

2. Ollen J (2006) A criterion-related validity test of selected indicators of musical sophistication using expert ratings [Doctoral thesis]: Ohio State University.
