# Supplementary material for: Syncopation, Body-Movement and Pleasure in Groove Music
Source: PLoS One. 2014 Apr 16;9(4):e94446. doi: 10.1371/journal.pone.0094446 (PMC3989225; doi:10.1371/journal.pone.0094446)
Supplement: Text S3 — Joint audio entropy. (DOCX) [file pone.0094446.s013.docx]

Supporting information Text S3

*Joint audio entropy*

Joint entropy, which is defined by the probability of more than one independent event occurring given a particular distribution model [[1](#_ENREF_1)], was calculated for the audio wave data. Thus, the joint entropy of the drum-breaks reflects the probability of each wave data sample occurring in relation to the distribution of data samples in the drum-break as a whole. For example, in rhythms with a fast and variable change in instrumentation, such as a drum-break with many onsets on different drums alternating frequently in complex and syncopated ways, the probability of a specific audio sample is low because it occurs rarely. The joint audio entropy of this drum-break is therefore high. Alternatively, in drum-breaks with few onsets on few drums alternating only rarely, the probability of a specific sample is higher, because it occurs more frequently in the audio. Such a drum-break would have low joint audio entropy. In this way, joint audio entropy represents the objective energy complexity of the audio input, and serves as a reasonable approximation to the event density measure used by Madison et al. [[2](#_ENREF_2)], although not confined to the sub-beat level. The MATLAB (2012, Mathworks, Inc.) function ‘Joint Entropy’ was used [[3](#_ENREF_3)], and measurements are reported in bits.

References

1. Temperley D (2007) Music and probability. Campridge, MA: MIT Press.

2. Madison G, Gouyon F, Ullén F, Hörnström K (2011) Modeling the tendency for music to induce movement in humans: First correlations with low-level audio descriptors across music genres. Journal of Experimental Psychology: Human Perception and Performance 37: 1578-1594.

3. Dwinnel W (2010) Joint entropy, MATLAB function. Available: <http://www.mathworks.com/matlabcentral/fileexchange/28695-joint-entropy>. Accessed 9 October 2013.
