# Supplementary material for: Syncopation, Body-Movement and Pleasure in Groove Music
Source: PLoS One. 2014 Apr 16;9(4):e94446. doi: 10.1371/journal.pone.0094446 (PMC3989225; doi:10.1371/journal.pone.0094446)
Supplement: Text S4 — Validation of syncopation index. (DOCX) [file pone.0094446.s014.docx]

Supporting information Text S4

*Validation of syncopation index*

A regression was performed in order to test whether our modified index of syncopation provided a better fit with ratings than the original index proposed by Longuet-Higgins and Lee [[1](#_ENREF_1)]. That is, whether an index that is sensitive to the instrumental configuration of drum-breaks is able to explain affective and embodied ratings more systematically than an index that treats all sonic information monophonically, regardless of instrumentation. Thus, we compared the variance explained by the two indices.

*Analysis*

For the monophonic indexing, the hihat was excluded from analysis, and the bass- and snare-drum were analysed as one instrumental layer. The exclusion of the hihat was necessary, since, due to its constant occurrence on the quaver pulse, it would cancel out all syncopations. First, regression was performed using a quadratic model, in which average ratings across each drum-break (N = 50) were regressed against syncopation values, once using the monophonic index, and once using the polyphonic index. Second, to test differences between the indexes more systematically, participants’ individual adjusted R^2^s from regressions using the monophonic and polyphonic indexes were compared using paired t-tests. All participants, regardless of musical background, were included in these tests (N = 66).

*Results*

Regression of mean ratings with degree of monophonic syncopation with a quadratic model yielded R^2^s of .2726 and .3064 for wanting to move and feelings of pleasure, respectively. With the polyphonic index, the R^2^ was .3565 for wanting to move, and .4304 for feelings of pleasure. Thus, compared to the polyphonic index, the R^2^s were smaller for the monophonic index, by .084 for wanting to move and by .124 for feelings of pleasure.

Paired t-tests comparing participants’ individual adjusted R^2^s showed that for wanting to move, the adjusted R^2^s using the polyphonic index (Mean = .1179, SE = .0134) were significantly higher than for the monophonic index (Mean = .098, SE = .0132) (*t*(65) = 3.26, *p* = .002). Similarly, for feelings of pleasure, the polyphonic index (Mean = .1322, SE = .0151) yielded significantly higher adjusted R^2^s than the monophonic index (Mean = .1172, SE = .0157) (*t*(65) = 2.94, *p* = .005). These results confirm that the polyphonic index of syncopation used in our study explains listeners’ embodied and affective responses to groove-based drum-breaks more consistently than the monophonic index of syncopation proposed by Longuet-Higgins and Lee [[1](#_ENREF_1)].

References

1. Longuet-Higgins HC, Lee C (1984) The rhythmic interpretation of monophonic music. Music Perception 1: 424-440.
