# Supplementary material for: Syncopation, Body-Movement and Pleasure in Groove Music
Source: PLoS One. 2014 Apr 16;9(4):e94446. doi: 10.1371/journal.pone.0094446 (PMC3989225; doi:10.1371/journal.pone.0094446)
Supplement: Text S5 — Controlling for ‘unusualness’ in experimenter-composed drum-breaks. (DOCX) [file pone.0094446.s015.docx]

Supporting information Text S5

*Controlling for ‘unusualness’ in experimenter-composed drum-breaks*

As reported, some of the drum-breaks were not ‘real’ drum-breaks but composed by the authors in order to increase the spread of syncopation at both ends of the spectrum. Since it is possible that these composed drum-breaks would be confounded with ‘unusualness’, due to their being constructed for the purpose of the experiment and not representing ecologically valid stimuli, we performed additional regressions using only ‘real’ drum-breaks.

*Analysis*

Only drum-breaks transcribed from the ‘real’ funk repertoire, as well as the two drum-breaks transcribed from Garageband were included (*N* = 36). The two Garageband drum-breaks were included here, since they sound like ‘real’ drum-breaks and are likely to elicit comparable responses. Regressions were performed separately for each rating question. First, we compared adjusted R^2^s for linear vs. quadratic models of average ratings, separately for each rating question. Second, we performed a one-tailed t-test on participants’ individual quadratic coefficients, also separately for wanting to move and pleasure.

*Results*

The regressions with average ratings using only ‘real’ drum-breaks showed that for movement ratings, the quadratic model yielded a better fit (*adjusted R^2^* = .0448) than the linear model (*adjusted R^2^ =* -.0158), although ANOVAs for neither model were significant (linear model *F*(1, 34) = .453, *p* = .505, quadratic model *F*(2, 33) = 1.82, *p* = .178). Pleasure ratings were also better explained with the quadratic model (*adjusted R2* = .2133) than the linear model (*adjusted R^2^* = .0777), and the quadratic model was significant (*F*(2, 33) = 5.74, *p* = .007), while the linear model was close-to-significant (linear model *F*(1, 34) = 3.95, *p* = .055). The one-tailed t-tests showed that mean coefficients were negative, both for wanting to move (Mean = -.000245, SE = .000430) and pleasure (Mean = -.000542, SE = .000050), but only significant for pleasure (*t(65) =* -10.916, *p* < .001), and not for wanting to move ((*t(65) =* -.570 *p* = .571).

These results suggest that, even when exclusively using ecologically valid drum-breaks, ratings of are better explained by an inverted U-shape than a linear model. However, the U-shape was, particularly for wanting to move, less robust than the original regressions using all drum-breaks. Thus, it is possible that the ‘unusualness’ of the experimenter-composed drum-breaks, which were purposefully composed to have very low and very high degrees of syncopation, significantly influenced ratings. In other words, they may have been rated as eliciting less pleasure and less wanting to move than drum-breaks with medium degree of syncopation not because of their low and high degrees of syncopation, but because of their artificial and less familiar structures. It can be argued that drum-breaks with very low and very high syncopation are simply *not groove* because they are difficult to find in the ‘real’ repertoire of grooves. Nonetheless, despite being less robust, the regressions with only ‘real’ drum-breaks were more U-shaped than linear. Thus, our findings at least support a trend in which medium degrees of syncopation optimise listeners’ wanting to move and experience of pleasure, regardless of whether the drum-breaks are ‘real’ or artificial.
