# Supplementary material for: Syncopation, Body-Movement and Pleasure in Groove Music
Source: PLoS One. 2014 Apr 16;9(4):e94446. doi: 10.1371/journal.pone.0094446 (PMC3989225; doi:10.1371/journal.pone.0094446)
Supplement: Text S6 — Effects of musical background on parabola vertex. (DOCX) [file pone.0094446.s016.docx]

Supporting information Text S6

*Effects of musical background on parabola vertex*

In addition to the analyses reported in the main article, we investigated effects of musical background by comparing the x coordinates of the vertex of the parabola fitted with a quadratic model of syncopation for the different groups. Such an analysis would, for example, indicate whether musicians preferred higher degrees of syncopation than non-musicians, when rating their maximum pleasure and wanting to move.

*Analysis*

Only vertices of participants whose adjusted R^2^ was greater for quadratic compared to linear modelling were included in this analysis. Number of participants was thus *N =* 48 for comparing vertices for wanting to move, and *N =* 45 for pleasure (still excluding the 9 participants who could not be categorised as musicians or non-musicians). Differences between groups were analysed using independent t-tests.

*Results*

The independent t-tests for x coordinates of the vertex for the quadratic parabola showed that for wanting to move, there were no significant differences between musicians (Mean = 35.24, SE = 1.71) and non-musicians (Mean = 31.15, SE = 1.89) (*t(46) =* 1.59*, p =* .119), nor between dancers (Mean = 34.08, SE = 1.59) and non-dancers (Mean = 32.22, SE = 2.19) (*t(46)* = .648*, p = .*520). There was a close-to-significant difference between groove-enjoyers (Mean = 35.30, SE = 1.56) and non-groove-enjoyers (Mean = 30.01, SE = 2.10) (*t(46) =* 2.00*, p =* .052). For experience of pleasure, there were no significant differences between groups on the x coordinate of the vertex, neither between musicians (Mean = 32.85, SE = 4.56) and non-musicians (Mean = 31.92, SE = 2.29) (*t(43)=* .164, *p* = .871), between dancers (Mean = 34.10, SE = 1.81) and non-dancers (Mean = 28.82, SE = 8.11) (*t(43) =* .879, *p* = .384), nor between groove-enjoyers (Mean = 31.63, SE = 3.52) and non-groove-enjoyers (Mean = 34.12, SE = 4.61) (*t(43) =* -.418, *p* = .678).

Thus, the preferred degree of syncopation during optimal desire to move and experience of pleasure was not significantly affected by musical training, dance experience nor groove familiarity, although a close-to-significant t-test suggests that those familiar with groove may prefer higher degrees of syncopation for wanting to move, compared to those less familiar with groove. While further research is required before confident conclusions can be drawn, it is possible that this trend could be explained by the sensitisation that may be present for groove enjoyers. That is, the more you listen to music associated with groove, the greater the complexity required for optimal embodied stimulation.
