# Supplementary material for: Correction: Syncopation, Body-Movement and Pleasure in Groove Music
Source: PLoS One. 2015 Sep 24;10(9):e0139409. doi: 10.1371/journal.pone.0139409 (PMC4581707; doi:10.1371/journal.pone.0139409)
Supplement: S1 Fig — (PDF) [file pone.0139409.s001.pdf]

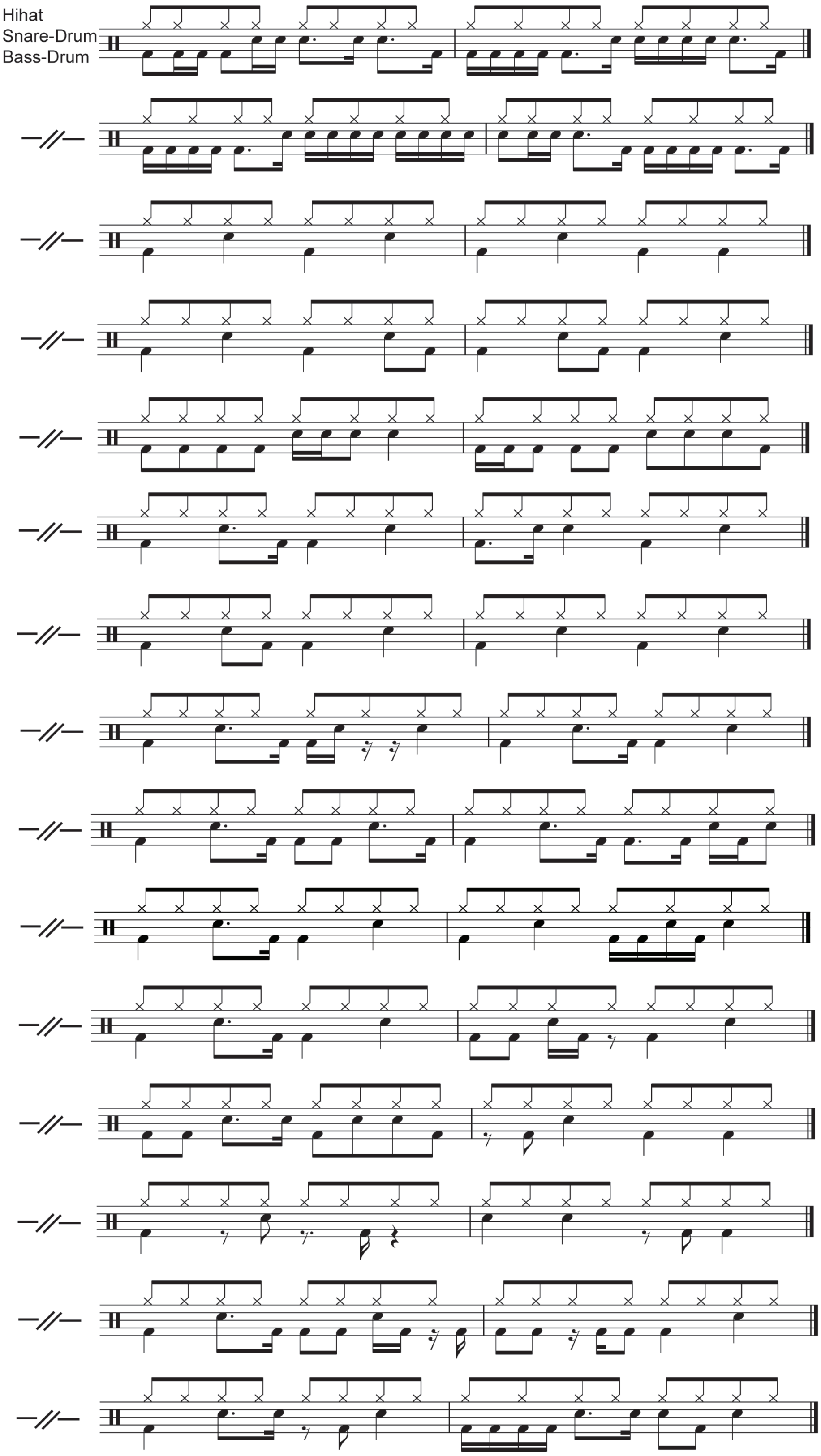

1. Experimenter-Composed Low no. 4
S: 0 JAE: 12.72 M: 2.27 P: 2.21

2. Experimenter-Composed Low no. 9
S: 0 JAE: 12.97 M: 1.85 P: 1.83

3. Experimenter-Composed Low no. 1
S: 6 JAE: 10.15 M: 2.68 P: 2.56

4. Fleetwood Mac: 'Prove Your Love'
S: 7 JAE: 10.60 M: 3.00 P: 2.80

5. Experimenter-Composed Low no. 6
S: 8 JAE: 12.17 M: 2.15 P: 2.30

6. Honeydrippers: 'Impeach the President'
S: 9 JAE: 10.44 M: 2.56 P: 2.48

7. Experimenter-Composed Low no. 3
S: 9 JAE: 10.24 M: 3.05 P:2.88

8. Annette Peacock: 'Survival'
S: 12 JAE: 10.59 M: 2.88 P: 2.76

9. Power of Zeus: 'Sorcerer of Isis'
S: 13 JAE: 11.92 M: 3.17 P: 2.97

10. Kool and the Gang: 'Jungle Jazz'
S: 14 JAE: 10.74 M: 2.92 P: 2.79

11. Gaz: 'Sing Sing'
S: 17 JAE: 10.94 M: 3.12 P: 3.11

12. Garageband Groove Template no. 1
S: 17 JAE: 11.26 M: 2.71 P: 2.76

13. Experimenter-Composed High no. 1
S: 18 JAE: 9.81 M: 2.05 P: 2.35

14. The Turtles: 'I'm Chief Kamanawalalea'
S: 19 JAE: 12.11 M: 3.20 P: 3.20

15. Orange Crush: 'Action'
S: 21 JAE: 11.05 M: 3.17 P: 3.03

Figure S1: Notational transcripts and audio descriptor values of drum-breaks no. 1–15. S = Syncopation degree (0 - 81). JAE = Joint audio entropy (9.81 - 13.64). M = Average ratings of wanting to move (1 - 5). P = Average ratings of pleasure (1 - 5).
