# Supplementary material for: Correction: Syncopation, Body-Movement and Pleasure in Groove Music
Source: PLoS One. 2015 Sep 24;10(9):e0139409. doi: 10.1371/journal.pone.0139409 (PMC4581707; doi:10.1371/journal.pone.0139409)
Supplement: S2 Fig — (PDF) [file pone.0139409.s002.pdf]

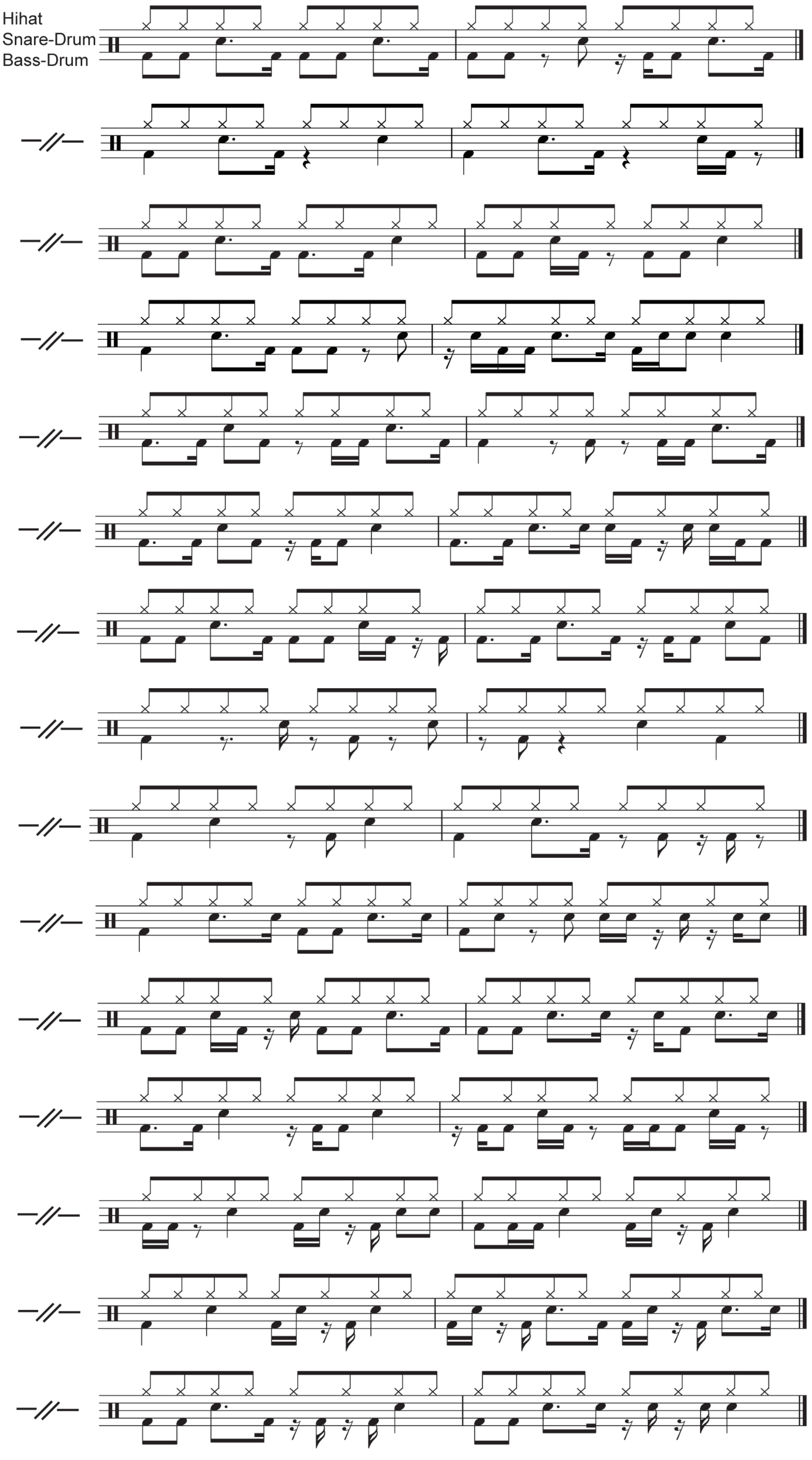


16. Donny Hathaway: ‘Magnificent Sanctuary Band’
S: 21 JAE: 12.19 M: 2.86 P: 2.83

17. Headhunters: ‘God Make Me Funky’
S: 22 JAE: 10.34 M: 3.18 P:3.17

18. Roy Ayers: ‘The Boogie Back’
S: 23 JAE: 11.94 M: 3.09 P: 2.94

19. Funk Inc.: ‘Kool is Back’
S: 24 JAE: 11.29 M: 2.76 P: 2.77

20. Gladys Knight and the Pips: ‘Who is She (and What is She to You)’
S: 24 JAE: 11.76 M: 2.95 P: 3.02

21. The Soul Searchers: ‘Ashley’s Roachclip’
S: 25 JAE: 12.35 M: 3.17 P: 3.05

22. Michael Jackson: ‘Ain’t No Sunshine’
S: 26 JAE: 12.79 M: 3.12 P: 3.18

23. Experimenter-Composed High no. 7
S: 27 JAE: 9.93 M: 2.06 P: 2.44

24. Please: ‘Sing a Simple Song’
S: 29 JAE: 10.60 M: 3.21 P: 3.12

25. Ike and Tina Turner: ‘Cussin’ and Cryin’ and Carryin’ On’
S: 32 JAE: 11.18 M: 3.23 P: 3.14

26. Garange Band Template no. 2
S: 34 JAE: 12.41 M: 3.45 P: 3.35

27. Pleasure: ‘Boundy Lady’
S: 36 JAE: 12.02 M: 3.17 P: 3.09

28. The Five Stairsteps and Cubie: ‘Don’t Change Your Love’
S: 37 JAE: 11.41 M: 3.12 P: 3.05

29. Captain Sky: ‘Super Sperm’
S: 37 JAE: 11.59 M: 3.08 P: 3.03

30. Assagai: ‘Telephone Girl’
S: 40 JAE: 11.67 M: 3.17 P: 2.98

Figure S2: Notational transcripts and audio descriptor values of drum-breaks no. 16–30. S = Syncopation degree (0 - 81). JAE = Joint audio entropy (9.81 - 13.64). M = Average ratings of wanting to move (1 - 5). P = Average ratings of pleasure (1 - 5).
