# Supplementary material for: Correction: Syncopation, Body-Movement and Pleasure in Groove Music
Source: PLoS One. 2015 Sep 24;10(9):e0139409. doi: 10.1371/journal.pone.0139409 (PMC4581707; doi:10.1371/journal.pone.0139409)
Supplement: S3 Fig — (PDF) [file pone.0139409.s003.pdf]

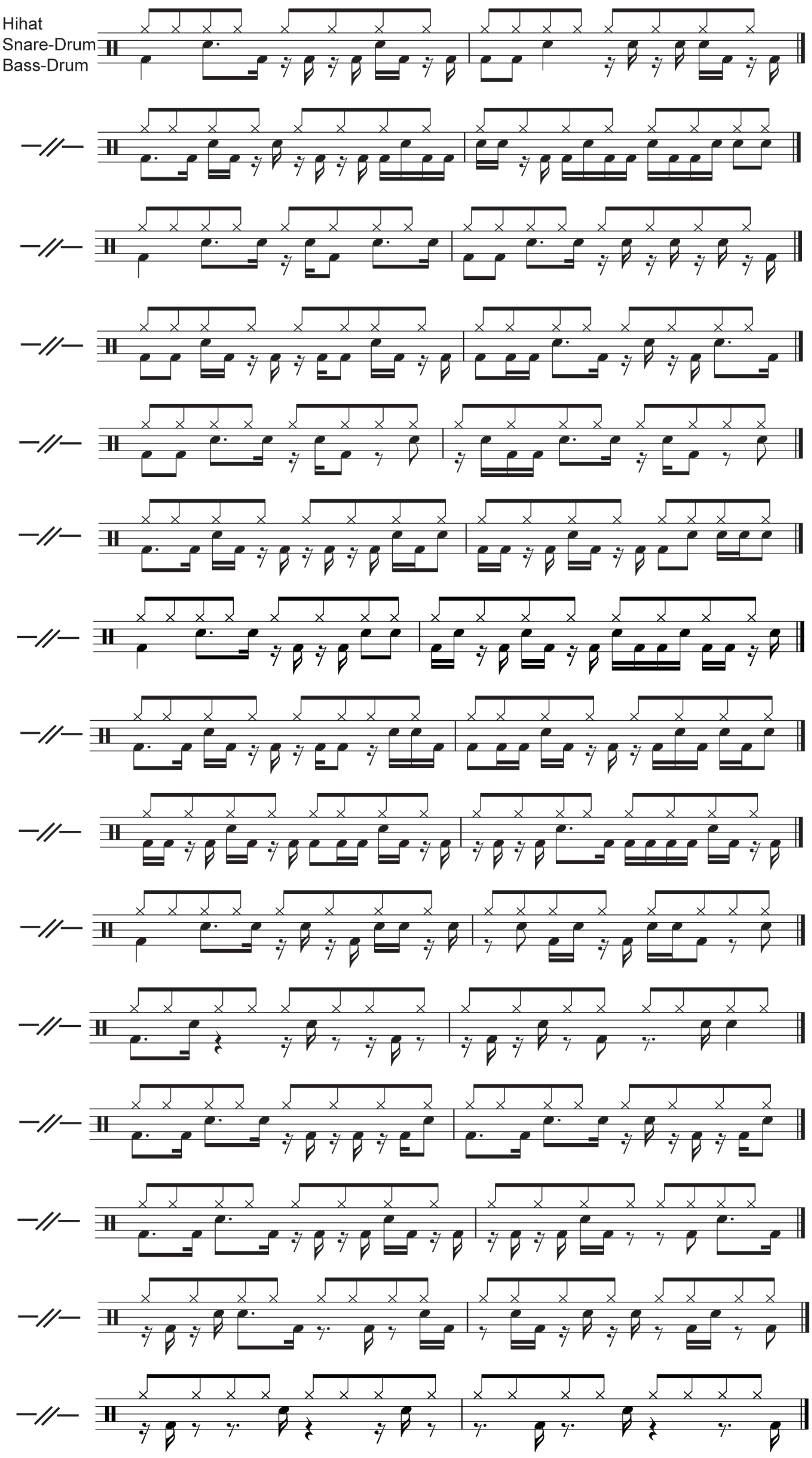

31. Funkadelic: ‘Good Old Music’
S: 43 JAE: 12.32 M: 3.21 P: 3.20

32. The Meters: ‘Here Come the Metermen’ (b)
S: 45 JAE: 13.07 M: 2.45 P: 2.91

33. Lou Donaldson: ‘Ode to Billy Joe’
S: 45 JAE: 11.72 M: 3.26 P: 3.21

34. Monk Higgins: ‘One Man Band (Plays All Alone)’
S: 46 JAE: 13.07 M: 3.52 P:3.32

35. The Winston’s: ‘Amen Brother’
S: 49 JAE: 11.54 M: 2.76 P: 2.88

36. The Meters: ‘Hey! Last Minute’ (a)
S: 52 JAE: 12.95 M: 3.18 P: 3.06

37. The Meters: ‘Here Come the Metermen’ (a)
S: 53 JAE: 12.45 M: 3.09 P: 3.12

38. The Meters: ‘Hey! Last Minute’ (b)
S: 53 JAE: 13.34 M: 2.91 P: 2.91

39. Love: ‘Doggone’ (b)
S: 54 JAE: 13.64 M: 3.26 P: 3.11

40. Al Green: ‘I’m Glad You’re Mine’
S: 54 JAE: 11.42 M: 2.55 P: 2.82

41. Experimenter-Composed High no. 3
S: 58 JAE: 10.63 M: 1.94 P: 2.24

42. I Gres: ‘Restless’
S: 60 JAE: 12.29 M: 2.89 P: 2.94

43. Love: ‘Doggone’ (a)
S: 60 JAE: 12.57 M: 3.23 P: 3.12

44. Experimenter-Composed High. no. 4
S: 62 JAE: 11.68 M: 1.98 P: 2.12

45. Experimenter-Composed High no. 8
S: 64 JAE: 10.20 M: 1.82 P: 2.23

Figure S3: Notational transcripts and audio descriptor values of drum-breaks no. 31–45. S = Syncopation degree (0 - 81). JAE = Joint audio entropy (9.81 - 13.64). M = Average ratings of wanting to move (1 - 5). P = Average ratings of pleasure (1 - 5).
