# Supplementary material for: Correction: Syncopation, Body-Movement and Pleasure in Groove Music
Source: PLoS One. 2015 Sep 24;10(9):e0139409. doi: 10.1371/journal.pone.0139409 (PMC4581707; doi:10.1371/journal.pone.0139409)
Supplement: S7 Fig — (PDF) [file pone.0139409.s005.pdf]

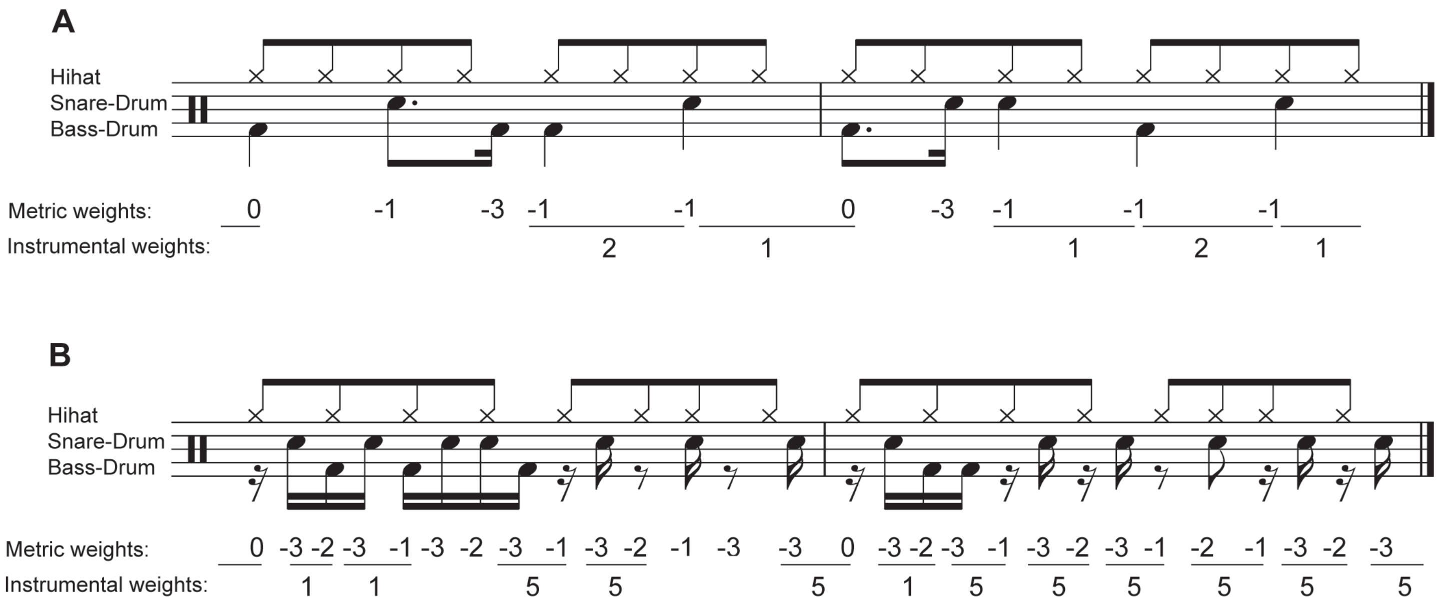

Figure S7: Drum-breaks of A: ‘Impeach the President’ by Honeydrippers (1973) with syncopation degree = 9, and B: ‘Actual Proof’ by Herbie Hancock (1974), with syncopation degree = 68.
