# Supplementary material for: Correction: Syncopation, Body-Movement and Pleasure in Groove Music
Source: PLoS One. 2015 Sep 24;10(9):e0139409. doi: 10.1371/journal.pone.0139409 (PMC4581707; doi:10.1371/journal.pone.0139409)
Supplement: S1 Table — (DOCX) [file pone.0139409.s006.docx]

Supporting information Table S1

**Table S1. Descriptive statistics for three-level categorisation of syncopation**

| **Predictors** | | **N (stimuli)** | **Mean index value** | **Minimum** | **Maximum** |
| --- | --- | --- | --- | --- | --- |
| Syncopation | Low | 17 | 12.53 | 0 | 22 |
|  | Medium | 17 | 33.71 | 23 | 46 |
|  | High | 16 | 61.25 | 49 | 81 |
| Joint Audio Entropy | Low | 17 | 10.48 | 9.81 | 11.18 |
|  | Medium | 17 | 11.71 | 11.26 | 12.29 |
|  | High | 16 | 12.78 | 12.31 | 13.64 |
