# Supplementary material for: Correction: Syncopation, Body-Movement and Pleasure in Groove Music
Source: PLoS One. 2015 Sep 24;10(9):e0139409. doi: 10.1371/journal.pone.0139409 (PMC4581707; doi:10.1371/journal.pone.0139409)
Supplement: S2 Text — (DOCX) [file pone.0139409.s007.docx]

Supporting information Text S2

*Index of syncopation*

The index used to measure the degree of syncopation of the drum-breaks was a modified version of the monophonic index developed by Longuet-Higgins and Lee [[1](#_ENREF_1)]. The modifications regarded the model of metre used, and the addition of instrumental weights to accommodate for the drum-breaks’ polyphonic character. A less hierarchical model of metre was used, compared to that of Longuet-Higgins and Lee (which is a purely theoretical model, corresponding to that of Lerdahl and Jackendoff [[2](#_ENREF_2)]), since empirical research has not been able to confirm a strictly hierarchical organisation of perceptual metre in rhythm [[3](#_ENREF_3),[4](#_ENREF_4)]. The model used is loosely based on our recent research [[4](#_ENREF_4)], investigating the perceptual effects of polyphonic context, instrumentation and metric location on syncopation in music. Supporting information Figure S5 depicts the model, which differs from that of Longuet-Higgins and Lee’s [[1](#_ENREF_1)] in its equal weights for all crotchet values, apart from the downbeat. Of all crotchets in a 4/4 meter, the downbeat is the only one shown consistently to be more salient than the other crotchets in empirical research [[3-5](#_ENREF_3)]. The addition of instrumental weights to the index was also a result of our study [[4](#_ENREF_4)], which showed that, in a drum-kit context, degree of syncopation depended on instrumentation. That is, if one instrument is syncopated while another instrument remains constant on the underlying pulse, then the degree of syncopation depends on the specific difference of instrumentation between the syncopated note and the underlying note on the pulse. Based on the study, we propose the following definition of syncopation:

*If N is a note that precedes a rest, R, and R has a metric weight greater than or equal to N, then the pair (N, R) is said to constitute a monophonic syncopation. If N is a note on a certain instrument that precedes a note on a different instrument, N_di_, and N_di_ has a metric weight greater than or equal to N, then the pair (N, N_di_) is said to constitute a polyphonic syncopation.*

The first part is taken from Longuet-Higgins and Lee’s [[1](#_ENREF_1)] own definition for monophonic syncopation, while the latter part is our new addition for the purpose of including polyphonic syncopations. Building on this definition, an index was developed according to which the degree of syncopation could be calculated for polyphonic drum-kit patterns. The index is described in Box 1.

**INDEX OF POLYPHONIC SYNCOPATION**

**Step 1**: Assign metric weights to each metric location, using the designated model of metric salience

**Step 2**: Locate instances of polyphonic syncopations.

**Step 3**: Assign instrumental weights: Three-stream syncopated bass-drum = 2, three-stream syncopated snare-drum = 1, two-stream syncopation = 5.

**Step 4**: Calculate degree of syncopation:

S = N – N_di_ + I

… where S is the degree of syncopation, N is the metric weight of the first note, N_di_ is the metric weight of the following note of different instrumentation and I is the instrumental weight of the syncopation.

Box 1. Index of polyphonic syncopation.

In rare instances in which a two-stream syncopation is followed by a hihat with greater or equal metric weight compared with the preceding hihat of the two-stream syncopation, a ‘double’ syncopation occurs in which the degree of syncopation equals the instrumental weight of 5 plus the difference in weight between the two hihats (the hihat following the two-stream syncopation and the hihat belonging to the two-stream syncopation).

As an example of how the index works, consider the rhythmic pattern in supporting information Figure S6, which is the drum-break in ‘Lifetime Monologue’ by Lou Rawls (1968). In step 1, metric weights are assigned to each note at every metric location in the pattern, according to the model of metric salience in supporting information Figure S5. The weights range between -3 and 0, and the downbeats of each bar are always given the highest value (0). The lowest value, -3, is given to every semiquaver location. The remaining metric locations are assigned metric weights according to the model in the same fashion. In step 2, all instances of polyphonic syncopation are located, according to the definition above: where a note N on a certain instrument precedes a note N_di_ on a different instrument and N_di_ has a metric weight greater than or equal to N. In supporting information Figure S6, these instances are underlined.

Next, the instrumental weights are assigned in step 3. The instrumental weight values are assigned to *pairs* of notes in the syncopation, as opposed to single events, since the instrumental weight depends on the relationship between the instruments in the pair. According to our previous research [[4](#_ENREF_4)], syncopation degree depends both on the type and number of instrumental streams involved, thus the index reflects these dependencies directly. The index only considers instrumental configurations relevant for the drum-breaks used in the study. For example, two-stream syncopations in which the bass-drum and snare-drum are syncopated against each other only are not indexed, since the hihat was kept constant on the quaver pulse in the drum-breaks and ensured that all syncopations always involved the hihat on the pulse. In all, only three instrumental configurations are indexed: Three-stream syncopations in which the bass-drum is syncopated against the snare-drum and hihat on the pulse are given an instrumental weight of 2 (i.e. bass-drum at metric location N followed by snare-drum and hihat at metric location N_di_, where N is metrically weaker than or equal to N_di_). Three-stream syncopations in which the snare-drum is syncopated against the bass-drum and hihat on the pulse, are given an instrumental weight of 1 (i.e. snare-drum at metric location N followed by bass-drum and hihat at metric location N_di_, where N is metrically weaker than or equal to N_di_). For two-stream syncopations, with the hihat on the pulse and either bass- or snare-drum on the syncopated note, the instrumental weight is 5. The instrumental weights are added below the underlined pairs of metric weights that constitute syncopations in Figure S6.

In the fourth and final step, the degree of syncopation is calculated for each instance of syncopation by adding the values of the instrumental weights to the values of difference in metric weights. For the red ringed syncopation in supporting information Figure S6, which is a two-stream syncopation, this means subtracting the metric weight of the snare-drum from the metric weight of the hihat and adding the instrumental weight of two-stream syncopations: -1 − (-3) + 5. Thus, the degree of syncopation here is 7. For the blue ringed syncopation, which is a three-stream syncopated snare-drum with the bass-drum and hihat on the pulse, the metric weight of the snare-drum is subtracted from that of the bass-drum, before adding the instrumental weight of a three-stream syncopated snare-drum with the pulse in the bass-drum and hihat: -2 − (-3) + 1 = 2. The green ringed syncopation, which is a three-stream syncopated bass-drum with a pulse in the snare and hihat, has a value of -1 − (-2) + 2 = 3. The remaining instances of syncopation have values of 3, 4, 3, 6, 3, 2, 4 and 4 respectively (the last instance being between the last note of the pattern and the first downbeat of its repetition). The sum of all the instances of syncopations represents the degree of syncopation for the rhythmic pattern as a whole. For ‘Lifetime Monologue’, this amounts to 41.

By way of comparison, supporting information Figure S7 presents two other rhythmic patterns, Figure S7.A from the drum-break of ‘Impeach the President’ by The Honeydrippers (1973) and Figure S7.B from ‘Actual Proof’ by Herbie Hancock (1974). When compared to ‘Lifetime Monologue’, ‘Impeach the President’ has considerably fewer instances of syncopation, and none that are in two-stream configurations. This is reflected in an overall syncopation degree of 9 for this pattern. ‘Actual Proof’, however, has considerably more syncopations, many of which occur in two-stream conditions, even on the downbeat, which is the most salient metric location. According to the polyphonic index of syncopation, the pattern has a relatively high value of syncopation: 68.

References

1. Longuet-Higgins HC, Lee C (1984) The rhythmic interpretation of monophonic music. Music Perception 1: 424-440.

2. Lerdahl F, Jackendoff R (1983) A generative theory of tonal music. Cambridge, Mass; London: MIT Press.

3. Ladinig O, Honing H, Haden G, Winkler I (2009) Probing attentive and preattentive emergent meter in adult listeners without extensive musical training. Music Perception 26: 377-386.

4. Witek MAG, Clarke EF, Kringelbach ML, Vuust P (in press) Effects of polyphonic context, instrumentation and metric location on syncopation in music. Music Perception.

5. Ladinig O (2009) Temporal expectations and their violations. [Doctoral thesis]: University of Amsterdam.
