# Supplementary material for: Correction: Syncopation, Body-Movement and Pleasure in Groove Music
Source: PLoS One. 2015 Sep 24;10(9):e0139409. doi: 10.1371/journal.pone.0139409 (PMC4581707; doi:10.1371/journal.pone.0139409)
Supplement: S4 Fig — (PDF) [file pone.0139409.s004.pdf]

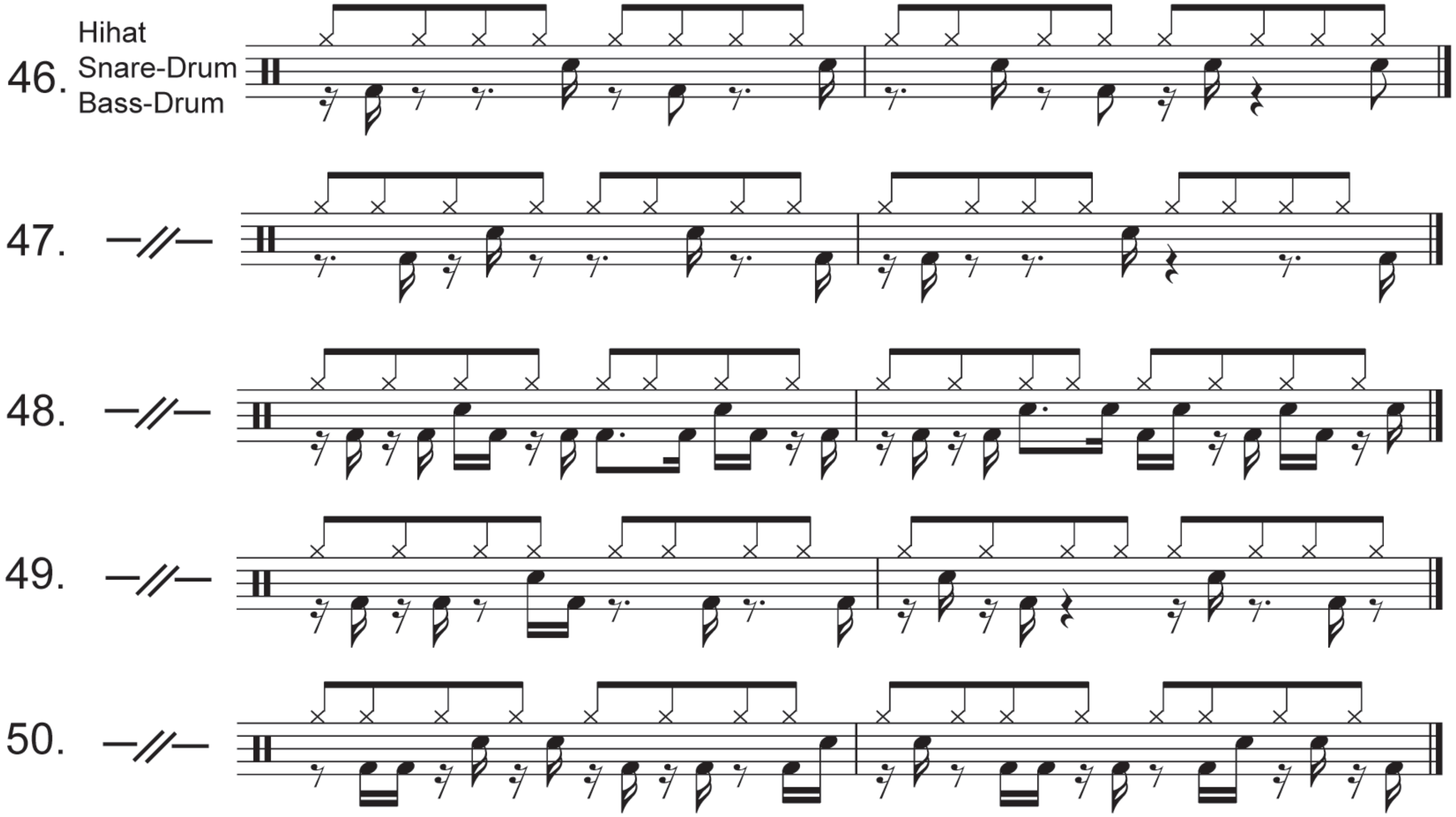

46. Experimenter-Composed High no. 6
S: 65 JAE: 10.25 M: 1.82 P: 2.03

47. Experimenter-Composed High no. 9
S: 66 JAE: 10.56 M: 1.77 P: 2.24

48. Love: 'Doggone' (c)
S: 71 JAE: 13.15 M: 3.08 P: 3.09

49. Experimenter-Composed High no. 5
S: 78 JAE: 11.44 M: 1.83 P: 2.05

50. Experimenter-Composed High no. 2
S: 81 JAE: 12.37 M: 2..00 P: 2.32

Figure S4: Notational transcripts and audio descriptor values of drum-breaks no. 46–50. S = Syncopation degree (0 - 81). JAE = Joint audio entropy (9.81 - 13.64). M = Average ratings of wanting to move (1 - 5). P = Average ratings of pleasure (1 - 5). JAE = Joint audio entropy.
